# Supplementary material for: Prevention of mother-to-child transmission of hepatitis B virus: protocol for a one-arm, open-label intervention study to estimate the optimal timing of tenofovir in pregnancy
Source: BMJ Open. 2020 Sep 13;10(9):e038123. doi: 10.1136/bmjopen-2020-038123 (PMC7488796; doi:10.1136/bmjopen-2020-038123)
Supplement: Author's manuscript [file bmjopen-2020-038123.draft_revisions.pdf]

BMJ Open

BMJ Open is committed to open peer review. As part of this commitment we make the peer review history of every article we publish publicly available.

When an article is published we post the peer reviewers' comments and the authors' responses online. We also post the versions of the paper that were used during peer review. These are the versions that the peer review comments apply to.

The versions of the paper that follow are the versions that were submitted during the peer review process. They are not the versions of record or the final published versions. They should not be cited or distributed as the published version of this manuscript.

BMJ Open is an open access journal and the full, final, typeset and author-corrected version of record of the manuscript is available on our site with no access controls, subscription charges or pay-per-view fees (http://bmjopen.bmj.com).

If you have any questions on BMJ Open's open peer review process please email info.bmjopen@bmj.com

BMJ Open

Prevention of mother-to-child transmission of hepatitis B virus: a one arm, open label intervention study to estimate the optimal timing of tenofovir in pregnancy, a study protocol

Journal:	*BMJ Open*
Manuscript ID	bmjopen-2020-038123
Article Type:	Protocol
Date Submitted by the Author:	28-Feb-2020
Complete List of Authors:	Bierhoff, Marieke; Shoklo Malaria Research Unit; Amsterdam UMC, Internal Medicine and Tropical Diseases<br>Nelson, Kenrad; Johns Hopkins University, Department of Epidemiology<br>Guo, Nan; Johns Hopkins University, Department of Anesthesiology<br>Jia, Yuanxi ; Johns Hopkins University, Department of Epidemiology<br>Angkurawaranon, Chaisiri ; Chiang Mai University, Department of Family Medicine<br>Jittamala, Podjanee ; Mahidol Oxford Tropical Medicine Research Unit; Mahidol University Faculty of Medicine Ramathibodi Hospital, Department of Tropical Hygiene<br>Carrara, Verena; Shoklo Malaria Research Unit, MCH<br>Watthanaworawit, Wanitda ; Shoklo Malaria Research Unit<br>Ling, Clare; Shoklo Malaria Research Unit; Oxford University, Centre for Tropical Medicine and Global Health<br>Tongprasert, Fuanglada; Chiang Mai University, Department of Obstetrics and Gynecology<br>van Vugt, Michele; Amsterdam UMC, Internal Medicine and Tropical Diseases<br>Rijken, Marcus; Utrecht University, Department of Obstetrics and Gynecology<br>Nosten, Francois; Oxford University, Centre for Tropical Medicine and Global Health; Oxford University, Centre for Tropical Medicine and Global Health<br>McGready, Rose; Shoklo Malaria Research Unit, Faculty of Tropical Medicine, Mahidol University; Oxford University, Centre for Tropical Medicine and Global Health<br>Ehrhardt, Stephan ; Johns Hopkins University, Department of Epidemiology<br>Thio, Chloe; Johns Hopkins University School of Medicine,
Keywords:	Public health < INFECTIOUS DISEASES, Tropical medicine < INFECTIOUS DISEASES, Fetal medicine < OBSTETRICS, Maternal medicine < OBSTETRICS, THERAPEUTICS, VIROLOGY

SCHOLARONE™
Manuscripts

I, the Submitting Author has the right to grant and does grant on behalf of all authors of the Work (as defined in the below author licence), an exclusive licence and/or a non-exclusive licence for contributions from authors who are: i) UK Crown employees; ii) where BMJ has agreed a CC-BY licence shall apply, and/or iii) in accordance with the terms applicable for US Federal Government officers or employees acting as part of their official duties; on a worldwide, perpetual, irrevocable, royalty-free basis to BMJ Publishing Group Ltd ("BMJ") its licensees and where the relevant Journal is co-owned by BMJ to the co-owners of the Journal, to publish the Work in this journal and any other BMJ products and to exploit all rights, as set out in our licence.

The Submitting Author accepts and understands that any supply made under these terms is made by BMJ to the Submitting Author unless you are acting as an employee on behalf of your employer or a postgraduate student of an affiliated institution which is paying any applicable article publishing charge ("APC") for Open Access articles. Where the Submitting Author wishes to make the Work available on an Open Access basis (and intends to pay the relevant APC), the terms of reuse of such Open Access shall be governed by a Creative Commons licence – details of these licences and which Creative Commons licence will apply to this Work are set out in our licence referred to above.

Other than as permitted in any relevant BMJ Author's Self Archiving Policies, I confirm this Work has not been accepted for publication elsewhere, is not being considered for publication elsewhere and does not duplicate material already published. I confirm all authors consent to publication of this Work and authorise the granting of this licence.

For peer review only

Prevention of mother-to-child transmission of hepatitis B virus: a one arm, open label intervention study to estimate the optimal timing of tenofovir in pregnancy, a study protocol

Marieke Bierhoff[1, 2], Kenrad E. Nelson[3], Nan Guo[4], Yuanxi Jia[3], Chaisiri Angkurawaranon[5], Podjanee Jittamala[6,7], Verena I Carrara[1], Wanitda Watthanaworawit[1], Clare L. Ling[1,8], Fuanglada Tongprasert[9], Michele van Vugt[2], Marcus J. Rijken[10], Francois Nosten[1,8], Rose McGready[1,8], Stephan Ehrhardt[3*], Chloe L. Thio[11*]

[1]Shoklo Malaria Research Unit, Mahidol-Oxford Tropical Medicine Research Unit, Faculty of Tropical Medicine, Mahidol University, Mae Sot, Thailand

[2]Department of Internal Medicine and Tropical Diseases, Academic Medical Center Amsterdam, the Netherlands

[3] Department of Epidemiology, Johns Hopkins Bloomberg School of Public Health, Baltimore, MD, USA

[4] Department of Anesthesiology, Perioperative and Pain Medicine, Stanford University, Stanford, CA, USA

[5] Department of Family Medicine, Faculty of Medicine, Chiang Mai University

[6]Mahidol-Oxford Tropical Medicine Research Unit, Faculty of Tropical Medicine, Mahidol University, Bangkok, Thailand

[7] Department of Tropical Hygiene, Faculty of Tropical Medicine, Mahidol University, Bangkok, Thailand

[8] Centre for Tropical Medicine and Global Health, Nuffield Department of Medicine, University of Oxford, Oxford, UK

[9] Department of Obstetrics and Gynecology, Faculty of Medicine, Chiang Mai University

[10]Department of Obstetrics and Gynecology, Utrecht University Medical Centre, Utrecht, Netherlands

[11] Department of Medicine, Johns Hopkins University School of Medicine, Baltimore, MD, USA

For peer review only

Corresponding author: Stephan Ehrhardt, Department of Epidemiology and Internal Medicine, Johns Hopkins University, School of Medicine 855 N Wolfe Street, 5th floor, Baltimore, MD 21205, e-mail: sehrhar6@jhu.edu

* Contributed equally

Word count: 3,337

Keywords: HBV, Antiviral therapy, maternal health

For peer review only

ABSTRACT

Introduction: Hepatitis B Virus (HBV) remains a public health threat and the main route of transmission is from mother to child (MTCT). Tenofovir disoproxil fumarate (TDF) treatment can reduce MTCT of HBV although the optimal timing to attain undetectable HBV DNA concentrations prior to delivery is unknown. This protocol describes the procedures following early initiation of maternal TDF prior to 20 weeks gestation to determine the efficacy, safety and feasibility of this approach in a limited-resource setting.

Methods and analyses: One hundred and seventy pregnant women from the Thailand-Myanmar border between 12 to <20 weeks gestational age will be enrolled into a one arm, open label, TDF treatment study with cessation of TDF one month after delivery. Sampling will occur monthly prenatal, at birth and at 1, 2, 4 and 6 months post-partum. Measurement of tenofovir concentrations in maternal and cord plasma is anticipated in 10-15 women who have detectable HBV DNA in their plasma at delivery and matched to 20-30 women who do not have detectable HBV DNA. Infant HBsAg status will be determined at 2 months of age and HBV DNA confirmed in HBsAg positive cases. Adverse events including risk of flare and adherence based on pill count and questionnaire will be monitored. Infants will receive HBV vaccinations at birth, 2, 4 and 6 months and hepatitis B immunoglobulin at birth if the mother is hepatitis B e antigen positive. Infant growth and neurodevelopment at 6 months will be compared to established local norms.

Ethics and dissemination: This study has ethical approval by the Ethics Committee of the Faculty of Tropical Medicine, Mahidol University (FTM ECF-019-06), Johns Hopkins University (IRB No: 00007432), Chiang Mai University (FAM-2559-04227), Oxford Tropical Research Ethics Committee (OxTREC Reference: 49-16) and by the local Tak Community Advisory Board (TCAB-02/REV/2016).

For peer review only

ARTICLE SUMMARY

Strengths and limitations of this study

1. This prospective cohort study will provide evidence towards the optimal time to initiate tenofovir disoproxil fumarate (TDF) treatment in pregnant woman to reduce Hepatitis B virus (HBV) DNA to undetectable concentrations (defined as <100 IU/ml) prior to birth, which is currently unknown.
2. Strategies for prevention of mother to child transmission (MTCT) of Human Immunodeficiency Virus (HIV) are not immediately transferable to HBV so the feasibility of implementing the recommended treatment for HBV in pregnant women in a limited-resource setting is overdue.
3. Challenges to adherence and safety in a context of low literacy where two thirds of pregnant women have limited education will be identified.
4. TDF during pregnancy for prevention of MTCT is a potential alternative to circumvent provision of hepatitis B immunoglobulins (HBIG) which remains out of reach for most limited-resource settings.
5. TDF in addition to HBIG and vaccination in infants born to hepatitis B e antigen positive mothers is the most comprehensive way to prevent MTCT but such a design does not provide information on the proportion of MTCT in women with an undetectable HBV DNA without HBIG.

For peer review only

INTRODUCTION

Background and rationale

In 2016 it is estimated that only 1 in 10 of the 257 million people living with hepatitis B virus (HBV) were aware of their infection and of those, 1 in 7 were on treatment, which is less than 2% of the HBV infections globally [1]. The current sustainable development goals aim to reduce the prevalence of HBV by 90% in 2030 [2]. The only rapid route to reach this goal is prevention of mother to child transmission (MTCT) of HBV the most common source of infection. Pregnancy provides a window of opportunity in the life of an adult to advocate for a screening intervention, raising awareness of HBV infection, potentially resulting in positive impacts on prevalence. Unfortunately both screening and prevention efforts are impeded by lack of access to universal health care and essential health services, a problem relevant to half of the world's population [3].

Maternal HBV infection is an important communicable disease on the Myanmar-Thailand border [4] and in other parts of Asia, the western Pacific and African Region, where more than 6% of adults are infected [1]. In antenatal clinics at the Shoklo Malaria Research Unit (SMRU) clinics, the hepatitis B surface antigen (HBsAg) prevalence is 6.4-8.3% with a hepatitis B e antigen (HBeAg) prevalence of 32.7% in those positive for HBsAg [4, 5]. Mothers who are HBeAg positive are at the highest risk of transmitting HBV to their infants, which is the basis for specifically targeting this group for neonatal hepatitis B immunoglobulins (HBIG) within 72 hours of life. In infants born to HBeAg positive mothers and provided HBIG and HBV vaccine, prevention of MTCT fails in an estimated 8–32% of cases [6]. Overall, there are two reasons to find alternatives to HBIG: i) failure to prevent MTCT in infants born in HBeAg positive women, and ii) for the 50% of the world's population without universal health care who lack access to HBIG as it remains a specialized product that is costly, requires a cold chain and has a restrictive prescription window to be effective [7].

The usage of antivirals for HIV in limited-resource settings has shown remarkable scale up and success and is a vital component of antenatal care programs [8]. Antivirals that are active against HBV like tenofovir disoproxil fumarate (TDF) may reduce the risk of MTCT by reducing the HBV DNA across gestation to a minimum at the time of delivery when MTCT typically occurs and might be a more feasible option than HBIG in a limited-resource setting [9]. The optimal gestational age to commence TDF is critical to know in

For peer review only

order to ensure HBV DNA is as low as possible prior to birth [10]. The precise threshold of HBV DNA, in the absence of HBIG that prevents MTCT is also still unknown. Initially, a HBV DNA of ≥6 log10 IU/ml was associated with a higher risk of MTCT, suggesting women in this category were the most important to target for antiviral therapy [11-13]. Although a maternal HBV DNA <6 log10 IU/ml is thought to be lower risk for MTCT the studies reporting this provided the infants with HBIG and vaccination. Although provision of TDF 300 mg daily in the third trimester of pregnancy significantly reduced perinatal transmission (OR, 0.10; 95% CI, 0.01 to 0.77; p=0.03) [6, 14-16], a mean of 8.6 ± 0.5 weeks of TDF prior to delivery resulted in a mean HBV DNA at delivery of 4 log 10 IU/ml with 32% of mothers having HBV DNA >5.3 log 10 (or 200,000) IU/ml [6]. This amount of HBV DNA is too high to forego HBIG, and if provided, may still not be sufficient to prevent infection. In the same study HBV DNA declined another log (median 3.52 log10 IU/ml) in the four weeks post-partum that TDF was provided. Gao *et al.,* showed that starting TDF treatment at an estimated gestational age of 24–27 weeks may achieve better suppression compared to starting in third trimester [16].

Existing data from trials involving non-pregnant patients are not informative due to differences in age, stage of HBV, infrequency of HBV DNA testing and because immune response and pharmacokinetics of TDF in pregnant women may differ from non-pregnant patients [17-20]. Pharmacokinetic studies have established that the area under the curve (AUC) and maximum drug concentration (C_{max}) of TDF are decreased in the second and third trimester of pregnancy, and clearance is increased[18, 21-24]. In a systematic review of TDF pharmacokinetics in pregnancy, none of the manuscripts reported reaching the TDF C_{max} of 300 ng/ml the efficacy threshold determined in non-pregnant HBV infected women [23]. These data suggest that TDF dosage and treatment initiation that are accurate for HIV and non-pregnant HBV infected patients may not be accurate for pregnant HBV infected women.

While some of the safety issues have been resolved with the use of TDF for prevention of MTCT of HIV, gaps remain regarding HBV [25, 26]. The main barriers to implementation of TDF are a lack of knowledge by the women of hepatitis B, their infectious status and the preventive strategies [27], poor adherence to the prophylactic drug regimen [9], and potential hepatitis flares after post-partum drug discontinuation [28], both of which need to be addressed in limited-resource settings where HBV is highly endemic [29].

The aim of this prospective interventional study is to determine the appropriate time in pregnancy to initiate TDF treatment of HBV in order to prevent MTCT. The viral kinetics of HBV DNA in pregnant women

For peer review only

receiving daily oral TDF commenced before 20 weeks gestation will be measured monthly. MTCT will be assessed at 2 months post-partum. While the study population is pregnant women attending antenatal and birth services on the Myanmar-Thailand border, the study has worldwide application to women who are pregnant and HBV positive. Moreover, we will study safety, feasibility and potential barriers to implementation of TDF in routine antenatal care (ANC) in a limited-resource setting.

Objectives

The primary objective is to estimate the time to HBV DNA suppression <2 log10 IU/ml (or <100 IU/ml) in HBV DNA positive women who start TDF late in the first or early in the second trimester.

Secondary objectives include: estimation of the proportion of women with HBV DNA <2 log10 IU/ml (or <100 IU/ml) at delivery; the concentration of TDF in maternal and cord blood at birth in women who have detectable HBV DNA and a subset of women with undetectable HBV DNA; to monitor the safety of TDF on the Myanmar-Thailand border in pregnant women with low health literacy rates [30] and address potential barriers to implementing TDF in early pregnancy to prevent MTCT of HBV; to determine the rate of hepatic flares post-partum after cessation of TDF; to estimate the proportion of cases of vertical transmission in infants of 2 months of age; to assess fetal growth by monthly ultrasound, and infant growth at 1, 2 ,4 and 6 months and neurodevelopment at 6 months.

Study design

This is a prospective, one arm, open label, intervention study. Women who enroll in the study will be provided with a daily 300 mg dose of oral TDF starting at enrollment until one month after delivery. Follow-up will continue at 1, 2, 4 and 6 months post-partum. A small sub-group of women are expected to require ongoing treatment for HBV flares and they will be followed for a further 6-9 months and provided drug therapy if required.

METHODS AND ANALYSIS

Setting

The study will be set in the clinics of Shoklo Malaria Research Unit (SMRU) on the border of Thailand and Myanmar (Figure 1). Since the 1990s the migrant influx from Myanmar to Thailand has increased for economic and political reasons, and to date there are approximately 3.5 million migrants in Thailand [31].

For peer review only

Over 25% of the migrant workers have no work permits and no legal status in Thailand [32]. Migrants are highly mobile, and the majority do not have access to basic health care which, combined with scarce services in Myanmar, put them at increased risk when seeking health services. In rural areas, labour migrants may be paid daily wages below Thailand's legal minimum wage reducing health-seeking behaviors which incur cost [33].

SMRU has provided humanitarian health care for marginalized populations for 30 years. One of the original objectives was to reduce malaria related maternal mortality from *Plasmodium falciparum* with screening embedded into routine maternal and child health care. The study will invite participants from three clinic sites (Mawker Thai, Wang Pha and Maela) serving marginalized populations (Figure 1). The long experience of SMRU working with the population has resulted in a high level of trust in the services. The maternal and child health services are in rural areas, can be provided in Karen and Burmese language, and is without costs [5, 34, 35].

At the time of study protocol submission (August 2017) antiviral treatment of HBV in pregnancy was not provided by the government in Myanmar or in Thailand, although it could be obtained by out of pocket expenditure beyond the means of this marginalized border population.

Study recruitment and enrollment

Pregnant women aged 16-49 years with a singleton viable pregnancy, an HBV infection, and an estimated gestational age of 12 to < 20 weeks determined by ultrasound will be counseled about the disease and invited to participate in the study. Part of this information session will include counseling on HBV in general, the need for adherence to the study drug and benefits and risks of the treatment. If a woman is interested, a blood sample will be collected to determine if she meets the criteria for enrollment i.e. HBeAg positive or HBV DNA >1.9 log 10 (>85 IU/mL). Women will be informed of the results at the next visit and be enrolled and started on TDF if all inclusion criteria are met (Table 1).

Follow up

Medical and Obstetric Data

Vital signs (blood pressure, tympanic temperature, heart rate, respiratory rate, fetal heart rate); weight, height, age, symphysis fundal height, fetal gestational age and heartbeat; medical history review (including

For peer review only

medications, vitamins and allergies), ethnicity, details of any past pregnancies, complications, place of delivery and outcome will be recorded; and physical examination completed (**Figure 2 and Table 2**).

Laboratory tests

A maternal whole blood sample obtained by venipuncture will be collected at study entry, monthly until delivery, at birth and at two post-partum visits (Figure 2). Maternal HBV DNA, alanine amniotransferase (ALT) and creatinine will be measured at all scheduled visits for the mother. Serum phosphate will be measured at baseline and at birth.

Monthly visits during pregnancy

From the time TDF commence, the pregnant women will return for a monthly study visit. Obstetric follow-up at each monthly visit will include vital signs, weight, symphysis fundal height measurement and fetal heart beat and concomitant medications will be recorded into the CRF. Fetal growth will be measured monthly by ultrasound (head circumference, abdominal circumference and femur length).

Adverse events (AE) will be reviewed, and reported as AE or serious AE (SAE) according to the problem. Compliance will be monitored by pill counting and a questionnaire on adherence.

Delivery

Pregnancy complications and mode of delivery will be recorded in the CRF. Anthropometric measurements of the infant will be done in the first hour of life and will include birth weight, length, head and arm circumference, by trained staff and according to standard operating procedures.

Vaccination: HBIG (if mother is HBeAg positive) and standard HBV vaccine (at birth and 2, 4 and 6 months of age) will be provided to the infants according to the Expanded Program on Immunization (EPI) [36-38].

At birth a 12 ml sample of cord blood, 6 ml into EDTA and 6 ml into a plain tube, will be collected for analysis of HBV DNA and tenofovir level and HBsAg reflecting the HBV status of the infant. Note this cord blood sample might not be collected if the woman is referred for caesarean section into the Public Hospital system.

A maternal serum sample will be used to measure ALT, creatinine, phosphorus, HBsAg (HBeAg only if the woman was HBeAg positive at baseline) and tenofovir level.

For peer review only

Post-partum follow-up

TDF will be continued until one month post-partum to decrease the risk of flares as the maternal immune system returns to baseline. All women will continue the study until the 4 month post-partum visit. At the 1 and 4 months post-partum visits ALT will be measured. If ALT concentrations are increased but there is not a true flare follow up will continue and repeat ALT testing done at 6 months. If the flare worsens or does not resolve, they will be placed on anti-HBV therapy and be reviewed by a physician.

All infants will be followed at 1, 2, 4 and 6 months. The infant will be screened for HBV at 2 months. At each visit infant anthropometry will be measured (body weight, length, head circumference) and feeding recorded. At 6 months neurodevelopment will be assessed. At month 2, 4 and 6 the infant will receive follow up HBV vaccinations.

Sample size

We calculated the sample size required to estimate the proportion of pregnant women expected to be undetectable at delivery with precision.

$$\boldsymbol{n} = \frac{(\mathbf{1.96})^2 \boldsymbol{p}(\mathbf{1} - \boldsymbol{p})}{\boldsymbol{d}^2}$$

There is no prior knowledge to predict the proportion of pregnant women expected to be undetectable at delivery, so we used the most conservative estimate. When this proportion is 50%, the required sample size is the largest. Based on a 95% confidence interval, a margin of error d = 8% and a conservative estimation of proportion p = 0.5, a sample size of n = 150 is required. We aim for n = 170 to allow for attrition.

Data analyses

HBV Viral kinetics

The proportion of women with undetectable HBV DNA at delivery will be reported with 95% confidence interval in all women and by high vs low HBV DNA groups. Median and interquartile range (IQR) of HBV DNA at baseline and each visit will be reported. Then HBV DNA will be logarithmically transformed and the median HBV DNA at baseline and at delivery will be compared using paired t-tests. A Generalized Estimating Equation (GEE) model will be used to assess whether baseline HBV DNA and adherence to TDF are associated with the logarithmically transformed HBV DNA over time. The Kaplan-Meier method will be

For peer review only

used to estimate the median time from initiation of TDF to an HBV DNA <100 IU/ml including all women and then, based on the distribution of HBV DNA, stratified by high vs low HBV DNA groups. A proportional hazard model will be constructed to assess the hazard ratio of reaching undetectable HBV DNA adjusting for the baseline HBV DNA concentration and adherence to TDF (# pills actually taken/# pills that should have been taken). For missing HBV DNA data, depending on the missingness mechanism, imputations using Multiple Imputations by Chained Equations (MICE) will be used. Sensitivity analyses will then be performed comparing analyses using complete and imputed data sets. Additional analyses may be conducted to further explore the data.

Adherence

The main analysis to determine adherence will be calculating percent adherence using pill counting data (# pills actually taken/# pills that should have been taken). As a confirmation of adherence, we will also measure TDF concentration in all women with detectable HBV DNA at delivery and in a matched subset of women who become undetectable for comparison (n=2x the number of women who do not become undetectable). This is anticipated to include 10-15 women who are HBV DNA detectable at delivery and matched to 20-30 women who are undetectable. This analytic sub-sample will be matched on age and initial DNA concentration or, if a matched pair analysis is not feasible because no "matching partners" exist, we will correct for these factors using multivariate regression methods.

The 'Adherence Starts with Knowledge' questionnaire ASK-12 is a tool to determine adherence and is derived from the ASK-20 questionnaire [39]. The questionnaire consists of 12 items related to drug adherence in three scales "forgetfulness", "treatment beliefs", and "behavior". Although reports demonstrating the usefulness of ASK-12 for patients, this tool has not been validated in the study population [40, 41]. Local pilot testing has resulted in a culturally appropriate version of this questionnaire and personnel will be trained to use it. We will use pill counts (gold standard) as well as TDF blood concentration (confirmatory) to validate this tool. The results will be analysed by first comparing differences among the groups, one-way analysis of variance and Bonferroni's multiple comparison tests.

Hepatic Flare

Although TDF is thought to be safe and acceptable in pregnancy, after discontinuation it might lead to hepatitis flares. There are conflicting data about the proportion and severity of flares after discontinuing

For peer review only

antiviral therapy [42-44]. There are no data in pregnant women regarding flares with longer duration of treatment and lower HBV DNA at delivery, nor is there information about predisposing characteristics to provide recommendations either before, or even after flares start. We will determine the proportion of woman who have a moderate (ALT>2.5x upper limit of normal) or severe (ALT>5x upper limit of normal) hepatitis flare after discontinuation of TDF [45]. We will estimate the impact of potential risk factors such as age, number of prior pregnancies, etc. for moderate or severe flares by including treatment duration and HBV DNA concentration at study start and at delivery into multivariable regression models.

Secondary analyses

We will screen for HBV infection by screening for HBsAg in infants at 2 months of age. If HBsAg is positive presence of HBV DNA from the same blood sample will be confirmed. If MCTC of HBV exists, the time of TDF initiation, maternal baseline HBV DNA, and TDF adherence will be compared between mothers with MTCT and those without using t-test and Chi-square test/Fisher's exact test. Infant anthropometry will be reported at 1, 2, 4 and 6 months. We will compare the infant anthropometry to the same age infant in general population. Additional analyses such as correlation between TDF use time and infant anthropometry will be conducted based on the data we observe.

ETHICS AND DISSEMINATION

Ethical considerations

SMRU has worked with pregnant women for three decades and provided research results that have had significant local and international impact [30, 46]. Maternal and infant mortality have decreased significantly over the years SMRU has worked with the population. Women come to deliver with SMRU by choice. These marginalized populations and their ability to negotiate the health landscape may be limited by education level but this is recognized by local health staff who are from the same community and form part of the research team [30, 46]. All communication is conducted in the preferred language of the patient and additional efforts are made to assist non-literate women. We ensure that participation is voluntary and that it is clearly understood that participation in the study can be ceased at any time without effect to the care provided. Extra precautions to inform participants about discontinuation while under TDF are also part of the information sheets.

For peer review only

The Tak Community Advisory Board (T-CAB) reviewed the project for relevance, advice and acceptability in a meeting in relation to this study prior to submission of the protocol [47]. Pertinent responses from the T-CAB in relation to this study were positive but also included a request to include adolescent pregnant women. Several T-CAB members discussed further the issue of consent and assent in relation to SMRU staff, pregnant women, study participants and guardians to further inform the study team on how this issue is best managed with the marginalized, mobile border population.

This study has been approved by the Ethics Committee of the Faculty of Tropical Medicine, Mahidol University (FTM ECF-019-06), Johns Hopkins University (IRB No: 00007432), Chiang Mai University (FAM-2559-04227), Oxford Tropical Research Ethics Committee (OxTREC Reference: 49-16) and by the local Tak Community Advisory Board (TCAB-02/REV/2016).

Informed consent

Trained counsellors will seek the mother's consent after the patient information sheet has been discussed. All queries from the mothers will be answered and those who agree will indicate consent by signing or providing thumbprints. All data collected will be treated confidentially and stored securely.

For peer review only

List of abbreviations

AE	Adverse event
ANC	Antenatal Care
ASK	Adherence Starts with Knowledge
DNA	deoxyribonucleic acid
HBeAg	Hepatitis B e antigen
HBIG	Hepatitis B immunoglobulin
HBsAg	Hepatitis B surface antigen
HBV	Hepatitis B virus
LIC	Low-income country
MTCT	Mother to child transmission
SMRU	Shoklo Malaria Research Unit
TDF	Tenofovir Disoproxil Fumarate

For peer review only

Author Statements

KN, SE, CLT and RM designed the protocol; SE, KN and CLT obtained funding; RM, CLT, SE, FT, CA and MB obtained the Ethical approvals; RM and MB set up the clinical work; WW and CLL prepared laboratory testing; NG and YJ supported statistical considerations; PJ, VIC, FT, MvV and FN supported the design and clinical implementation of the study; all authors read and contributed to the present manuscript. All authors read and approved the final manuscript.

Data statement

After analyses of the data the data will be made available.

Patient and Public Involvement statement

The Tak Community Advisory Board (T-CAB) reviewed the project for relevance, advice and acceptability in a meeting in relation to this study prior to submission of the protocol. Pertinent responses from the T-CAB in relation to this study were positive but also included a request to include adolescent pregnant women. Several T-CAB members discussed further the issue of consent and assent in relation to SMRU staff, pregnant women, study participants and guardians to further inform the study team on how this issue is best managed with the marginalized, mobile border population.

Funding

The work is supported by the following awards: Thrasher Research Fund [grant number: TRF13096] to Johns Hopkins University; the Wellcome-Trust Major Overseas Program in Southeast Asia [grant number: 106698/Z/14/Z] to Mahidol University Oxford Tropical Medicine Research Programme which directly supports PJ, CLL, FN, RM from Shoklo Malaria Research Unit; and Chiang Mai University Thailand supporting MB, FT and CA.

Competing interests

The authors declare that they have no competing interests

Acknowledgements

We would kindly like to acknowledge all contributions of SMRU staff and doctors for their feedback in finalizing the research proposal. Also the data management team for contributing to the Case Report Form development and our quality control team, Moo Kho Paw and Rattanaporn Raksapraidee, and clinical trials support group, for their efforts. Myo Chit Min helped to design the map.

For peer review only

References

1. WHO. Guidelines for the prevention, care and treatment of persons with chronic hepatitis B infection. Geneva: World Health Organization; 2015. Report No.: NLM classification: WC 536.”. . Available at: https://www.who.int/hepatitis/publications/hepatitis-b-guidelines/en/. Accessed 19 December 2019.

2. Thomas DL. Global Elimination of Chronic Hepatitis. N Engl J Med **2019**; 380(21): 2041-50.

3. Bloom DE, Khoury A, Subbaraman R. The promise and peril of universal health care. Science (New York, NY) **2018**; 361(6404).

4. Banks T, Kang J, Watts I, et al. High hepatitis B seroprevalence and risk factors for infection in pregnant women on the Thailand-Myanmar Border. J Infect Dev Ctries **2016**; 10(4): 384-8.

5. Bierhoff M, Angkurawaranon C, Myat Min A, et al. Maternal Hepatitis B Infection Burden, Comorbidity and Pregnancy Outcome in a Low-Income Population on the Myanmar-Thailand Border: A Retrospective Cohort Study. J Pregnancy **2019**; 2019: 8435019.

6. Pan CQ, Duan Z, Dai E, et al. Tenofovir to Prevent Hepatitis B Transmission in Mothers with High Viral Load. N Engl J Med **2016**; 374(24): 2324-34.

7. Thio CL, Guo N, Xie C, Nelson KE, Ehrhardt S. Global elimination of mother-to-child transmission of hepatitis B: revisiting the current strategy. Lancet Infect Dis **2015**; 15(8): 981-5.

8. Lattof SR, Tuncalp O, Moran AC, et al. Developing measures for WHO recommendations on antenatal care for a positive pregnancy experience: a conceptual framework and scoping review. BMJ open **2019**; 9(4): e024130.

9. Li XM, Yang YB, Hou HY, et al. Interruption of HBV intrauterine transmission: a clinical study. World J Gastroenterol **2003**; 9(7): 1501-3.

10. Beigi R, Noguchi L, Parsons T, et al. Pharmacokinetics and placental transfer of single-dose tenofovir 1% vaginal gel in term pregnancy. J Infect Dis **2011**; 204(10): 1527-31.

11. Thilakanathan C, Wark G, Maley M, et al. Mother-to-child transmission of hepatitis B: Examining viral cut-offs, maternal HBsAg serology and infant testing. Liver international : official journal of the International Association for the Study of the Liver **2018**; 38(7): 1212-9.

For peer review only

12. Wen WH, Chang MH, Zhao LL, et al. Mother-to-infant transmission of hepatitis B virus infection: significance of maternal viral load and strategies for intervention. J Hepatol **2013**; 59(1): 24-30.

13. Lu Y, Zhu FC, Liu JX, et al. The maternal viral threshold for antiviral prophylaxis of perinatal hepatitis B virus transmission in settings with limited resources: A large prospective cohort study in China. Vaccine **2017**; 35(48 Pt B): 6627-33.

14. Jourdain G, Ngo-Giang-Huong N, Harrison L, et al. Tenofovir versus Placebo to Prevent Perinatal Transmission of Hepatitis B. New England Journal of Medicine **2018**; 378(10): 911-23.

15. Lee YS, Lee HS, Kim JH, et al. Role of tenofovir disoproxil fumarate in prevention of perinatal transmission of hepatitis B virus from mother to child: a systematic review and meta-analysis. The Korean journal of internal medicine **2019**.

16. Gao F, Zhang WT, Lin YY, Wang WM, Xu N, Bai GQ. Early Start Of Tenofovir Treatment Achieves Better Viral Suppression In Pregnant Women With A High HBV Viral Load: A Real-World Prospective Study. Infection and drug resistance **2019**; 12: 3475-84.

17. Thio CL, Guo N, Xie C, Nelson KE, Ehrhardt S. Global elimination of mother-to-child transmission of hepatitis B: revisiting the current strategy. The Lancet Infectious Diseases **2015**; 15(8): 981-5.

18. Colbers AP, Hawkins DA, Gingelmaier A, et al. The pharmacokinetics, safety and efficacy of tenofovir and emtricitabine in HIV-1-infected pregnant women. AIDS (London, England) **2013**; 27(5): 739-48.

19. Pyra M, Anderson PL, Hendrix CW, et al. Tenofovir and tenofovir-diphosphate concentrations during pregnancy among HIV-uninfected women using oral preexposure prophylaxis. AIDS (London, England) **2018**; 32(13): 1891-8.

20. Bierhoff M, Pinyopornpanish K, Pinyopornpanish K, et al. Retrospective Review of Documentation Practices of Hepatitis B Immunoglobulin, Birth Dose, and Vaccination at the Hospital of Birth, in Thai Nationals and Migrants in Northern Thailand. Open forum infectious diseases **2019**; 6(12): ofz518.

21. Best BM, Burchett S, Li H, et al. Pharmacokinetics of tenofovir during pregnancy and postpartum. HIV Med **2015**; 16(8): 502-11.

22. Benaboud S, Hirt D, Launay O, et al. Pregnancy-related effects on tenofovir pharmacokinetics: a population study with 186 women. Antimicrob Agents Chemother **2012**; 56(2): 857-62.

For peer review only

23. Bierhoff M, Smolders EJ, Tarning J, et al. Pharmacokinetics of oral tenofovir disoproxil fumarate in pregnancy and lactation: a systematic review. Antiviral therapy **2019**; 24(7): 529-40.

24. Cressey TR, Avihingsanon A, Halue G, et al. Plasma and Intracellular Pharmacokinetics of Tenofovir Disoproxil Fumarate 300 mg Every 48 Hours vs 150 mg Once Daily in HIV-Infected Adults With Moderate Renal Function Impairment. Clin Infect Dis **2015**; 61(4): 633-9.

25. Siberry GK, Jacobson DL, Kalkwarf HJ, et al. Lower Newborn Bone Mineral Content Associated With Maternal Use of Tenofovir Disoproxil Fumarate During Pregnancy. Clin Infect Dis **2015**; 61(6): 996-1003.

26. Van Dyke RB, Chadwick EG, Hazra R, Williams PL, Seage GR, 3rd. The PHACS SMARTT Study: Assessment of the Safety of In Utero Exposure to Antiretroviral Drugs. Front Immunol **2016**; 7: 199.

27. Yankam BM, Anye CS, Nkfusai NC, Shirinde J, Cumber SN. Knowledge and practice of pregnant women and health care workers on hepatitis B prevention in the Limbe and Muyuka health districts of the south west region of Cameroon. The Pan African medical journal **2019**; 33: 310.

28. Durante-Mangoni E, Vitrone M, Parrella A, et al. Efficacy and safety of tenofovir, entecavir, and telbivudine for chronic hepatitis B in heart transplant recipients. Transpl Infect Dis **2016**; 18(3): 319-25.

29. Suk-Fong Lok A. Hepatitis B Treatment: What We Know Now and What Remains to Be Researched. Hepatology communications **2019**; 3(1): 8-19.

30. Gilder ME, Moo P, Hashmi A, et al. "I can't read and don't understand": Health literacy and health messaging about folic acid for neural tube defect prevention in a migrant population on the Myanmar-Thailand border. PLoS One **2019**; 14(6): e0218138.

31. Manoyos V TA, Srithanaviboonchai K, Yangyuenkul S, Grimes RM. Sexual risk-behaviors for HIV infections among young cross-border migrant workers living in urban Chiang Mai, Thailand. Journal of Health Research **2016**; 30(5): 347-53.

32. International, Organization, for, Imigration. IOM Thailand National Strategy 2017-2020. Available at: https://thailand.iom.int/iom-thailand-national-strategy-2017-2020. Accessed June 2019.

33. Meyer SR, Decker MR, Tol WA, Abshir N, Mar AA, Robinson WC. Workplace and security stressors and mental health among migrant workers on the Thailand-Myanmar border. Social psychiatry and psychiatric epidemiology **2016**; 51(5): 713-23.

For peer review only

34. Culhane-Pera KA, Sriphetcharawut S, Thawsirichuchai R, Yangyuenkun W, Kunstadter P. Afraid of Delivering at the Hospital or Afraid of Delivering at Home: A Qualitative Study of Thai Hmong Families' Decision-Making About Maternity Services. Matern Child Health J **2015**; 19(11): 2384-92.

35. Suphanchaimat R, Putthasri W, Prakongsai P, Tangcharoensathien V. Evolution and complexity of government policies to protect the health of undocumented/illegal migrants in Thailand - the unsolved challenges. Risk Manag Healthc Policy **2017**; 10: 49-62.

36. WHO. Expanded Program on Immunization Factsheet Myanmar. Available at: http://www.searo.who.int/immunization/data/myanmar.pdf. Accessed December 2018.

37. Cutts FT, Claquin P, Danovaro-Holliday MC, Rhoda DA. Monitoring vaccination coverage: Defining the role of surveys. Vaccine **2016**; 34(35): 4103-9.

38. Schalm SW, Mazel JA, de Gast GC, et al. Prevention of hepatitis B infection in newborns through mass screening and delayed vaccination of all infants of mothers with hepatitis B surface antigen. Pediatrics **1989**; 83(6): 1041-8.

39. Matza LS, Park J, Coyne KS, Skinner EP, Malley KG, Wolever RQ. Derivation and validation of the ASK-12 adherence barrier survey. The Annals of pharmacotherapy **2009**; 43(10): 1621-30.

40. Sasaki J, Kawayama T, Yoshida M, et al. Adherence barriers to inhaled medicines in Japanese older patients with asthma evaluated using the "Adherence Starts with Knowledge 20" (ASK-20) questionnaire. The Journal of asthma : official journal of the Association for the Care of Asthma **2019**; 56(6): 632-41.

41. Kimura Y, Koya T, Hasegawa T, et al. Characterization of low adherence population in asthma patients from Japan using Adherence Starts with Knowledge-12. Allergology international : official journal of the Japanese Society of Allergology **2020**; 69(1): 61-5.

42. Nguyen V, Tan PK, Greenup AJ, et al. Anti-viral therapy for prevention of perinatal HBV transmission: extending therapy beyond birth does not protect against post-partum flare. Aliment Pharmacol Ther **2014**; 39(10): 1225-34.

43. Xu WM, Cui YT, Wang L, et al. Lamivudine in late pregnancy to prevent perinatal transmission of hepatitis B virus infection: a multicentre, randomized, double-blind, placebo-controlled study. Journal of viral hepatitis **2009**; 16(2): 94-103.

44. Liem KS, Gehring AJ, Feld JJ, Janssen HLA. Challenges with stopping long-term nucleos(t)ide analogue therapy in patients with chronic hepatitis B. Gastroenterology **2019**.

For peer review only

45. Wang L, Kourtis AP, Ellington S, Legardy-Williams J, Bulterys M. Safety of tenofovir during pregnancy for the mother and fetus: a systematic review. Clin Infect Dis **2013**; 57(12): 1773-81.

46. Parker DM, Carrara VI, Pukrittayakamee S, McGready R, Nosten FH. Malaria ecology along the Thailand-Myanmar border. Malar J **2015**; 14: 388.

47. Pratt B, Lwin KM, Zion D, Nosten F, Loff B, Cheah PY. Exploitation and community engagement: can community advisory boards successfully assume a role minimising exploitation in international research? Dev World Bioeth **2015**; 15(1): 18-26.

For peer review only

Figure 1. Map of the central office of SMRU in Mae Sot, and the study sites Wang Pha (WPA), Mawker Thai (MKT) and Maela (MLA) on the Myanmar-Thailand border where the study will be conducted.

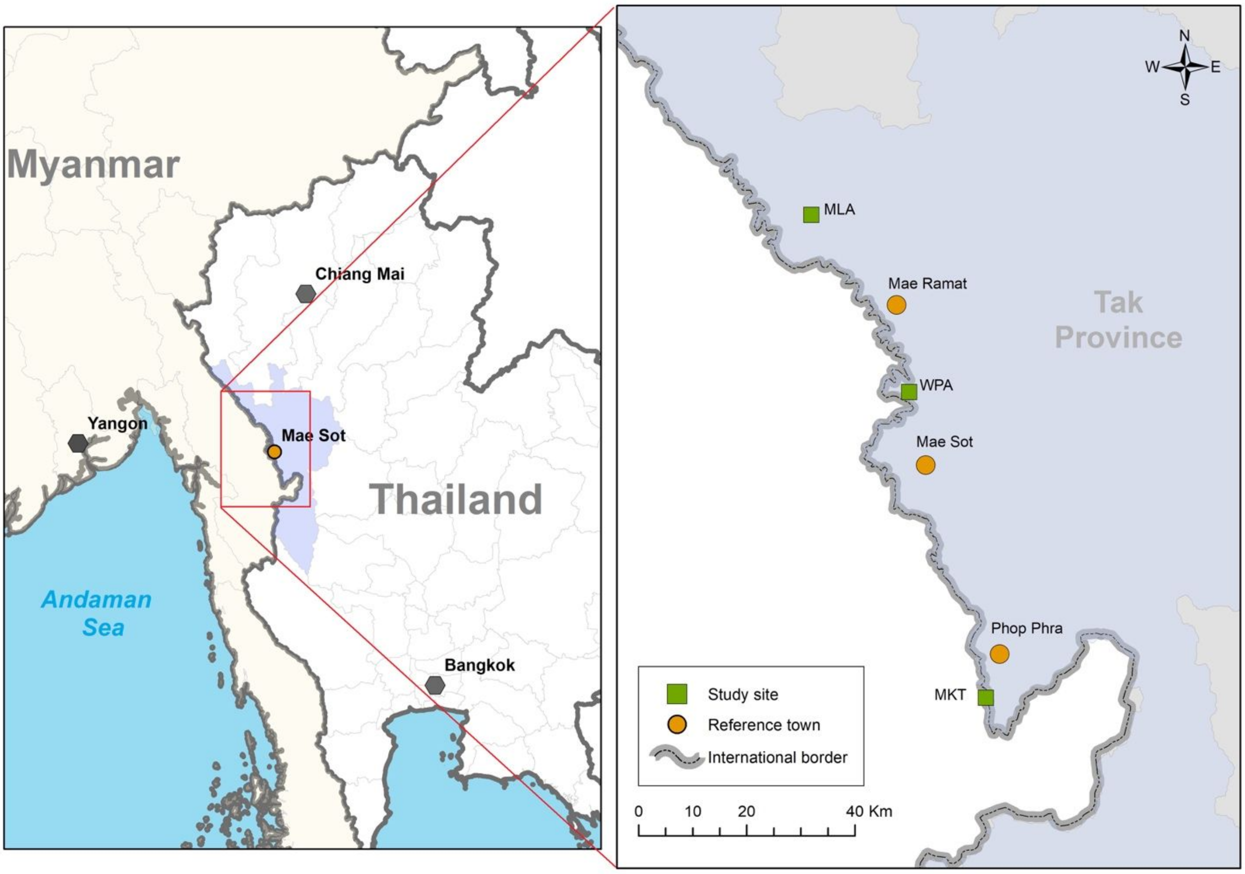

For peer review only

Figure 2. Schematic reproduction of the study design

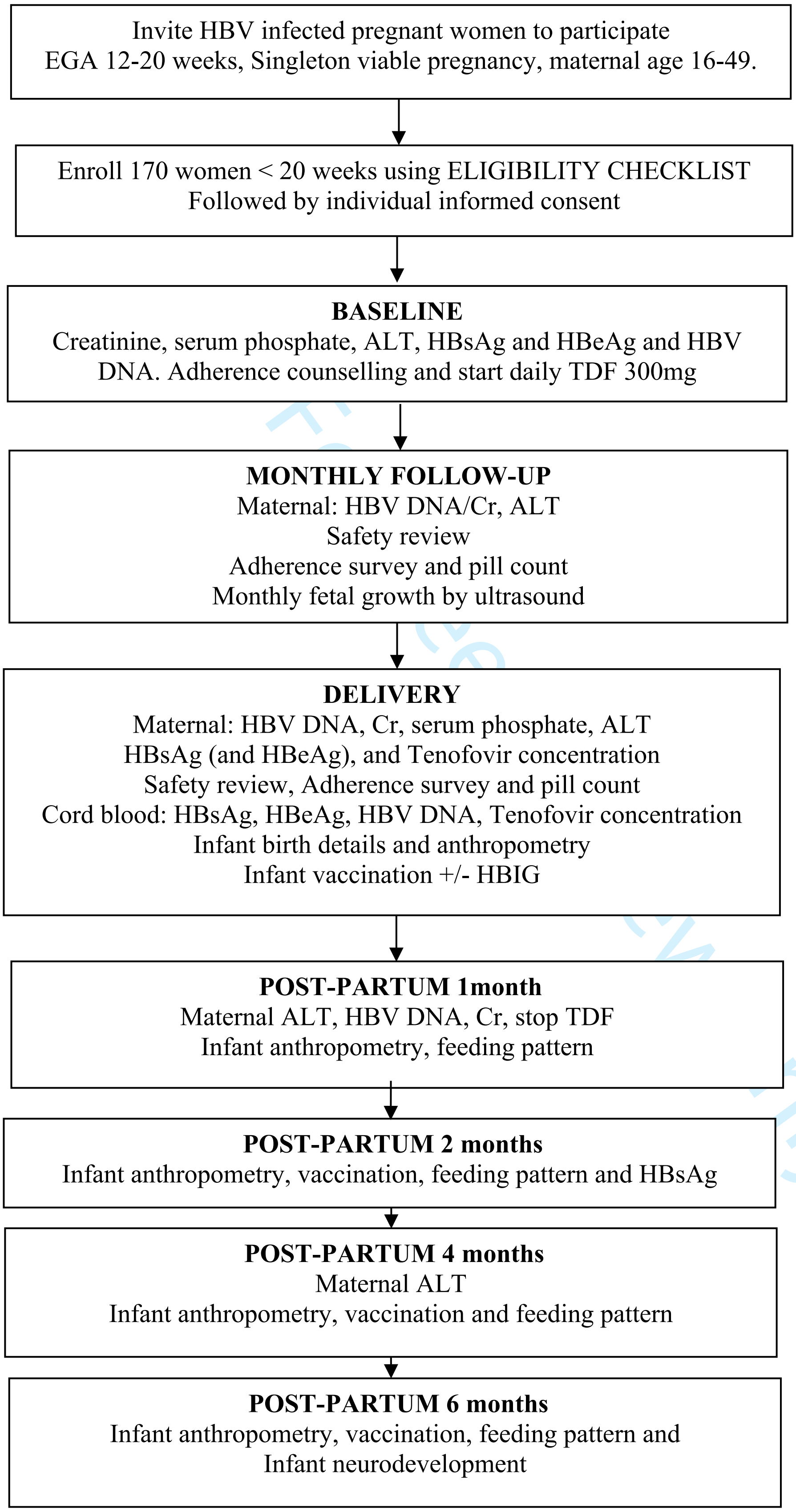

For peer review only

Table 1. Inclusion and exclusion criteria

Inclusion criteria	Exclusion criteria
Willing and able to give informed consent for participation in the study	Negative qualitative HBV DNA if HBeAg negative
Hepatitis B infected (HBsAg confirmed positive and qualitative HBV DNA positive in HBeAg negative mothers)	HIV positive or on immunosuppressive therapy for other illnesses
Female, 16-49 years	Elevated creatinine (>1 mg/dL)
EGA[1] 12-20 weeks at start TDF[2]	Abnormal serum phosphate (<2.4 and >4.5 mg/dL)
Willing to take TDF[2] daily during pregnancy	History of kidney disease
Plans to deliver at SMRU[3]	History of pregnancy complications

[1]Estimated Gestational Age, [2]Tenofovir Disoproxil Fumarate, [3]Shoklo Malaria Research Unit

Table 2. Overview of measurements and interventions per visit

Procedures	Visits timing						
	Start	Monthly visits	Delivery	1 month PP	2 months PP	4 months PP	6 months PP
		On TDF	On TDF	Stop TDF		End mother	End infant
Eligibility checklist	X						
Informed consent	X						
Ultrasound	X	X	X				
Demographics	X						
Medical history	X						
Physical examination	X	X	X	X		X	
**Laboratory tests**							
HBsAg/HBeAg	X						
HBV DNA	X	X	X	X			
Creatinine	X	X	X	X			
Serum Phosphate	X		X				
ALT	X	X	X	X		X[1]	
TDF Concentration			X[2]				
Cord HBsAg/HBV DNA			X[2]				

For peer review only

Infant HBsAg					X[4]		
Pill count		X	X	X			
Adverse event assessments		X	X	X			
Infant anthropometry			X	X	X	X	X
Infant neurodevelopment							X
Adherence survey		X	X	X			
Infant HB vaccination			X[3]		X	X	X

[1] Follow-up will continue if suspected or confirmed flare, [2] Cord blood sample, [3]If mother HBeAg positive also HBIG , [4] Venepuncture sample blood and only for HBsAg

For peer review only

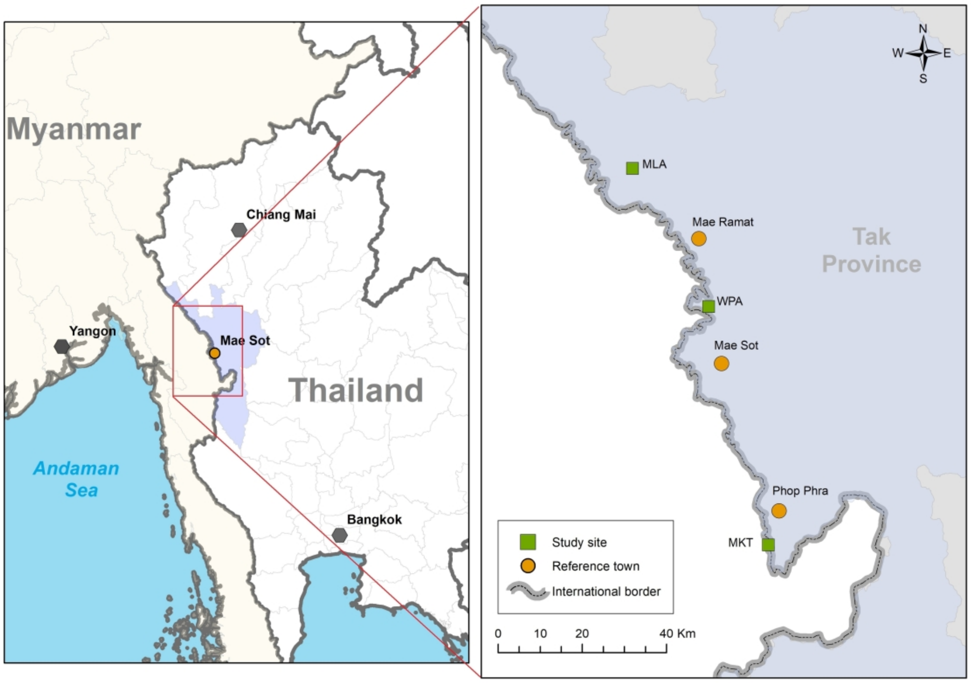

Figure 1. Map of the central office of SMRU in Mae Sot, and the study sites Wang Pha (WPA), Mawker Thai (MKT) and Maela (MLA) on the Myanmar-Thailand border where the study will be conducted.

296x210mm (300 x 300 DPI)

BMJ Open

Prevention of mother-to-child transmission of hepatitis B virus: a one arm, open label intervention study to estimate the optimal timing of tenofovir in pregnancy, a study protocol

Journal:	*BMJ Open*
Manuscript ID	bmjopen-2020-038123.R1
Article Type:	Protocol
Date Submitted by the Author:	08-Jun-2020

Complete List of Authors:	Bierhoff, Marieke; Shoklo Malaria Research Unit; Amsterdam UMC, Internal Medicine and Tropical Diseases<br>Nelson, Kenrad; Johns Hopkins University, Department of Epidemiology<br>Guo, Nan; Johns Hopkins University, Department of Anesthesiology<br>Jia, Yuanxi ; Johns Hopkins University, Department of Epidemiology<br>Angkurawaranon, Chaisiri ; Chiang Mai University, Department of Family Medicine<br>Jittamala, Podjanee ; Mahidol Oxford Tropical Medicine Research Unit; Mahidol University Faculty of Medicine Ramathibodi Hospital, Department of Tropical Hygiene<br>Carrara, Verena; Shoklo Malaria Research Unit, MCH<br>Watthanaworawit, Wanitda ; Shoklo Malaria Research Unit<br>Ling, Clare; Shoklo Malaria Research Unit; Oxford University, Centre for Tropical Medicine and Global Health<br>Tongprasert, Fuanglada; Chiang Mai University, Department of Obstetrics and Gynecology<br>van Vugt, Michele; Amsterdam UMC, Internal Medicine and Tropical Diseases<br>Rijken, Marcus; Utrecht University, Department of Obstetrics and Gynecology<br>Nosten, Francois; Oxford University, Centre for Tropical Medicine and Global Health; Oxford University, Centre for Tropical Medicine and Global Health<br>McGready, Rose; Shoklo Malaria Research Unit, Faculty of Tropical Medicine, Mahidol University; Oxford University, Centre for Tropical Medicine and Global Health<br>Ehrhardt, Stephan ; Johns Hopkins University, Department of Epidemiology<br>Thio, Chloe; Johns Hopkins University School of Medicine,

**Primary Subject Heading**:	Infectious diseases
Secondary Subject Heading:	Global health, Obstetrics and gynaecology, Public health, Reproductive medicine
Keywords:	Public health < INFECTIOUS DISEASES, Tropical medicine < INFECTIOUS DISEASES, Fetal medicine < OBSTETRICS, Maternal

	medicine < OBSTETRICS, THERAPEUTICS, VIROLOGY

SCHOLARONE™
Manuscripts

I, the Submitting Author has the right to grant and does grant on behalf of all authors of the Work (as defined in the below author licence), an exclusive licence and/or a non-exclusive licence for contributions from authors who are: i) UK Crown employees; ii) where BMJ has agreed a CC-BY licence shall apply, and/or iii) in accordance with the terms applicable for US Federal Government officers or employees acting as part of their official duties; on a worldwide, perpetual, irrevocable, royalty-free basis to BMJ Publishing Group Ltd ("BMJ") its licensees and where the relevant Journal is co-owned by BMJ to the co-owners of the Journal, to publish the Work in this journal and any other BMJ products and to exploit all rights, as set out in our licence.

The Submitting Author accepts and understands that any supply made under these terms is made by BMJ to the Submitting Author unless you are acting as an employee on behalf of your employer or a postgraduate student of an affiliated institution which is paying any applicable article publishing charge ("APC") for Open Access articles. Where the Submitting Author wishes to make the Work available on an Open Access basis (and intends to pay the relevant APC), the terms of reuse of such Open Access shall be governed by a Creative Commons licence – details of these licences and which Creative Commons licence will apply to this Work are set out in our licence referred to above.

Other than as permitted in any relevant BMJ Author's Self Archiving Policies, I confirm this Work has not been accepted for publication elsewhere, is not being considered for publication elsewhere and does not duplicate material already published. I confirm all authors consent to publication of this Work and authorise the granting of this licence.

For peer review only

Prevention of mother-to-child transmission of hepatitis B virus: a one arm, open label intervention study to estimate the optimal timing of tenofovir in pregnancy, a study protocol

Marieke Bierhoff[1, 2], Kenrad E. Nelson[3], Nan Guo[4], Yuanxi Jia[3], Chaisiri Angkurawaranon[5], Podjanee Jittamala[6,7], Verena I Carrara[1], Wanitda Watthanaworawit[1], Clare L. Ling[1,8], Fuanglada Tongprasert[9], Michele van Vugt[2], Marcus J. Rijken[10], Francois Nosten[1,8], Rose McGready[1,8], Stephan Ehrhardt[3*], Chloe L. Thio[11*]

[1]Shoklo Malaria Research Unit, Mahidol-Oxford Tropical Medicine Research Unit, Faculty of Tropical Medicine, Mahidol University, Mae Sot, Thailand

[2]Department of Internal Medicine and Tropical Diseases, Academic Medical Center Amsterdam, the Netherlands

[3] Department of Epidemiology, Johns Hopkins Bloomberg School of Public Health, Baltimore, MD, USA

[4] Department of Anesthesiology, Perioperative and Pain Medicine, Stanford University, Stanford, CA, USA

[5] Department of Family Medicine, Faculty of Medicine, Chiang Mai University

[6]Mahidol-Oxford Tropical Medicine Research Unit, Faculty of Tropical Medicine, Mahidol University, Bangkok, Thailand

[7] Department of Tropical Hygiene, Faculty of Tropical Medicine, Mahidol University, Bangkok, Thailand

[8] Centre for Tropical Medicine and Global Health, Nuffield Department of Medicine, University of Oxford, Oxford, UK

[9] Department of Obstetrics and Gynecology, Faculty of Medicine, Chiang Mai University

[10]Department of Obstetrics and Gynecology, Utrecht University Medical Centre, Utrecht, Netherlands

[11] Department of Medicine, Johns Hopkins University School of Medicine, Baltimore, MD, USA

For peer review only - http://bmjopen.bmj.com/site/about/guidelines.xhtml

Corresponding author: Stephan Ehrhardt, Department of Epidemiology and Internal Medicine, Johns Hopkins University, School of Medicine 855 N Wolfe Street, 5th floor, Baltimore, MD 21205, e-mail: sehrhar6@jhu.edu

* Contributed equally

Word count: 3,645

Keywords: HBV, Antiviral therapy, maternal health

For peer review only

ABSTRACT

Introduction: Hepatitis B Virus (HBV) remains a public health threat and the main route of transmission is from mother to child (MTCT). Tenofovir disoproxil fumarate (TDF) treatment can reduce MTCT of HBV although the optimal timing to attain undetectable HBV DNA concentrations prior to delivery is unknown. This protocol describes the procedures following early initiation of maternal TDF prior to 20 weeks gestation to determine the efficacy, safety and feasibility of this approach in a limited-resource setting.

Methods and analyses: One hundred and seventy pregnant women from the Thailand-Myanmar border between 12 to <20 weeks gestational age will be enrolled into a one arm, open label, TDF treatment study with cessation of TDF one month after delivery. Sampling will occur monthly prenatal, at birth and at 1, 2, 4 and 6 months post-partum. Measurement of tenofovir concentrations in maternal and cord plasma is anticipated in 10-15 women who have detectable HBV DNA at delivery and matched to 20-30 women with no detectable HBV DNA. Infant HBsAg status will be determined at two months of age and HBV DNA confirmed in HBsAg positive cases. Adverse events including risk of flare and adherence, based on pill count and questionnaire, will be monitored. Infants will receive HBV vaccinations at birth, 2, 4 and 6 months and hepatitis B immunoglobulin at birth if the mother is hepatitis B e antigen positive. Infant growth and neurodevelopment at six months will be compared to established local norms.

Ethics and dissemination: This study has ethical approval by the Ethics Committee of the Faculty of Tropical Medicine, Mahidol University (FTM ECF-019-06), Johns Hopkins University (IRB No: 00007432), Chiang Mai University (FAM-2559-04227), Oxford Tropical Research Ethics Committee (OxTREC Reference: 49-16) and by the local Tak Community Advisory Board (TCAB-02/REV/2016). The article will be published as an open access publication.

For peer review only - http://bmjopen.bmj.com/site/about/guidelines.xhtml

ARTICLE SUMMARY

Strengths and limitations of this study

1. This prospective cohort study will inform the optimal time to initiate tenofovir disoproxil fumarate (TDF) treatment in pregnant women to reduce Hepatitis B virus (HBV) DNA to undetectable concentrations (defined as <100 IU/ml) prior to birth, which is a strength since this is currently unknown.
2. Strategies for prevention of mother to child transmission (MTCT) of human immunodeficiency virus (HIV) are not immediately transferable to HBV so another strength is that this study investigates the feasibility of implementing the recommended treatment for HBV in pregnant women in a limited-resource setting.
3. It is important to identify challenges to adherence and safety in a context of low literacy where two thirds of pregnant women have limited education to ensure that potential implementation of this strategy is feasible
4. This study investigates TDF usage during pregnancy for prevention of MTCT as a potential alternative to hepatitis B immunoglobulin (HBIG), which remains too expensive and difficult to obtain in most limited-resource settings, and will inform future studies that forgo HBIG.
5. This study investigates TDF in addition to HBIG and vaccination in infants born to hepatitis B e antigen positive mothers as this is the most comprehensive way to prevent MTCT, but such a design does not provide information on the proportion of MTCT in women with an undetectable HBV DNA without HBIG.

For peer review only - http://bmjopen.bmj.com/site/about/guidelines.xhtml

INTRODUCTION

Background and rationale

In 2016 it was estimated that only 1 in 10 of the 257 million people living with hepatitis B virus (HBV) were aware of their infection and of those, 1 in 7 were on treatment, which is less than 2% of the HBV infections globally [1]. The current sustainable development goals aim to reduce the prevalence of HBV by 90% by 2030 [2]. An essential route to reach this goal is prevention of mother to child transmission (MTCT) of HBV, the most common source of infection. Pregnancy provides a window of opportunity in the life of an adult to advocate for a screening intervention, raising awareness of HBV infection, potentially resulting in positive impacts on prevalence. Unfortunately both screening and prevention efforts are impeded by lack of access to universal health care and essential health services, a problem relevant to half of the world's population [3].

Maternal HBV infection is an important communicable disease on the Myanmar-Thailand border [4] and in other parts of Asia, the western Pacific and African Region, where more than 6% of adults are infected [1]. In antenatal clinics at the Shoklo Malaria Research Unit (SMRU) clinics, the hepatitis B surface antigen (HBsAg) prevalence is 6.4-8.3% with a hepatitis B e antigen (HBeAg) prevalence of 32.7% in those positive for HBsAg [4, 5]. Mothers who are HBeAg positive are at the highest risk of transmitting HBV to their infants, which is the basis for specifically targeting this group for neonatal hepatitis B immunoglobulins (HBIG) within 72 hours of life. In infants born to HBeAg positive mothers and provided HBIG and HBV vaccine, prevention of MTCT fails in an estimated 8–32% of cases [6]. Overall, there are two reasons to find alternatives to HBIG: i) failure to prevent MTCT in infants born in HBeAg positive women, and ii) for the 50% of the world's population without universal health care who lack access to HBIG as it remains a specialized product that is costly, requires a cold chain and has a restrictive prescription window to be effective [7].

The usage of antivirals for HIV in limited-resource settings has shown remarkable scale up and success and is a vital component of antenatal care programs [8]. Antivirals that are active against HBV like tenofovir disoproxil fumarate (TDF) may reduce the risk of MTCT by reducing the HBV DNA across gestation to a minimum at the time of delivery when MTCT typically occurs and might be a more feasible option than HBIG in a limited-resource setting [9]. The optimal gestational age to commence TDF is critical to know to

For peer review only - http://bmjopen.bmj.com/site/about/guidelines.xhtml

ensure HBV DNA is as low as possible prior to birth [10]. The precise threshold of HBV DNA, in the absence of HBIG that prevents MTCT is also still unknown. Initially, a HBV DNA of ≥6 log10 IU/ml was associated with a higher risk of MTCT, suggesting women in this category were the most important to target for antiviral therapy [11-13]. Although a maternal HBV DNA <6 log10 IU/ml is thought to be lower risk for MTCT the studies reporting this provided the infants with HBIG and vaccination. Although provision of TDF 300 mg daily in the third trimester of pregnancy significantly reduced perinatal transmission (OR, 0.10; 95% CI, 0.01 to 0.77; p=0.03) [6, 14-16], a mean of 8.6 ± 0.5 weeks of TDF prior to delivery resulted in a mean HBV DNA at delivery of 4 log 10 IU/ml with 32% of mothers having HBV DNA >5.3 log 10 (or 200,000) IU/ml [6]. This amount of HBV DNA is too high to forego HBIG, and if provided, may still not be sufficient to prevent infection. In the same study HBV DNA declined another log (median 3.52 log10 IU/ml) in the four weeks post-partum that TDF was provided. Gao *et al.,* showed that starting TDF treatment at an estimated gestational age of 24–27 weeks may achieve better suppression compared to starting in third trimester [16].

Existing data from trials involving non-pregnant patients are not informative due to differences in age, stage of HBV, frequency of HBV DNA testing, and immune response and pharmacokinetics of TDF in pregnant women compared to non-pregnant patients [17-20]. Pharmacokinetic studies have established that the area under the curve (AUC) and maximum drug concentration (C_{max}) of TDF are decreased in the second and third trimester of pregnancy, and clearance is increased[18, 21-24]. In a systematic review of TDF pharmacokinetics in pregnancy, none of the manuscripts reported reaching the TDF C_{max} of 300 ng/ml the efficacy threshold determined in non-pregnant HBV infected women [23]. These data suggest that TDF dosage and treatment initiation that are accurate for HIV and non-pregnant HBV infected patients may not be accurate for pregnant HBV infected women.

While some of the safety issues have been resolved with the use of TDF for prevention of MTCT of HIV, gaps remain regarding HBV [25, 26]. The main barriers to implementation of TDF are a 1) lack of knowledge by the women of hepatitis B, of their infection status, and of prevention strategies [27], 2) poor adherence to the prophylactic drug regimen [9], and 3) potential for hepatitis flares after post-partum drug discontinuation [28]. All of these need to be evaluated and addressed in limited-resource settings where HBV is highly endemic [29].

For peer review only - http://bmjopen.bmj.com/site/about/guidelines.xhtml

The aim of this prospective interventional study is to determine the appropriate time in pregnancy to initiate TDF treatment of HBV in order to reach HBV DNA <100 IU/ml at delivery. Knowing this will allow future studies where TDF can be initiated at the appropriate time so that HBIG may not be needed to prevent MTCT of HBV. The viral kinetics of HBV DNA in pregnant women receiving daily oral TDF commenced before 20 weeks gestation will be measured monthly. Infants will receive HBIG and birth-dose monovalent HBV vaccine. MTCT will be assessed at two months post-partum. While the study population is pregnant women attending antenatal and birth services on the Myanmar-Thailand border, the study has worldwide application to women who are pregnant and HBV positive. Moreover, we will study safety, feasibility and potential barriers to implementation of TDF in routine antenatal care (ANC) in a limited-resource setting.

Objectives

The primary objective is to estimate the time to HBV DNA suppression <2 log10 IU/ml (or <100 IU/ml) in HBV DNA positive women who start TDF late in the first or early in the second trimester.

Secondary objectives include: estimation of the proportion of women with HBV DNA <2 log10 IU/ml (or <100 IU/ml) at delivery; the concentration of TDF in maternal and cord blood at birth in women who have detectable HBV DNA and a subset of women with undetectable HBV DNA; to monitor the safety of TDF on the Myanmar-Thailand border in pregnant women with low health literacy rates [30] and address potential barriers to implementing TDF in early pregnancy to prevent MTCT of HBV; to determine the rate of hepatic flares post-partum after cessation of TDF; to estimate the proportion of cases of vertical transmission in infants of two months of age; to assess fetal growth by monthly ultrasound, and infant growth at 1, 2 ,4 and 6 months and neurodevelopment at six months.

Study design

This is a prospective, one arm, open label, intervention study. Women who enroll in the study will be provided with a daily 300 mg dose of oral TDF starting at enrollment until one month after delivery. Follow-up will continue at 1, 2, 4 and 6 months post-partum. A small sub-group of women are expected to require ongoing treatment for HBV flares and they will be followed for a further 6-9 months and provided drug therapy if required.

For peer review only - http://bmjopen.bmj.com/site/about/guidelines.xhtml

METHODS AND ANALYSIS

Setting

The study will be set in the clinics of Shoklo Malaria Research Unit (SMRU) on the border of Thailand and Myanmar (Figure 1). Since the 1990s the migrant influx from Myanmar to Thailand has increased for economic and political reasons, and to date there are approximately 3.5 million migrants in Thailand [31]. Over 25% of the migrant workers have no work permits and no legal status in Thailand [32]. Migrants are highly mobile, and the majority do not have access to basic health care which, combined with scarce services in Myanmar, put them at increased risk when seeking health services. In rural areas, labour migrants may be paid daily wages below Thailand's legal minimum wage reducing health-seeking behaviors which incur cost [33].

SMRU has provided humanitarian health care for marginalized populations for 30 years. One of the original objectives was to reduce malaria related maternal mortality from *Plasmodium falciparum* with screening embedded into routine maternal and child health care. The study will invite participants from three clinic sites (Mawker Thai, Wang Pha and Maela) serving marginalized populations (Figure 1). The long experience of SMRU working with the population has resulted in a high level of trust in the services. The maternal and child health services are in rural areas, can be provided in Karen and Burmese language, and is without costs [5, 34, 35].

At the time of study protocol submission (August 2017) antiviral treatment of HBV in pregnancy was not provided by the government in Myanmar or in Thailand, although it could be obtained by out of pocket expenditure beyond the means of this marginalized border population.

Study recruitment and enrollment

Pregnant women aged 16-49 years with a singleton viable pregnancy, an HBV infection, and an estimated gestational age of 12 to < 20 weeks determined by ultrasound will be counseled about the disease and invited to participate in the study. Part of this information session will include counseling on HBV in general, the need for adherence to the study drug and benefits and risks of the treatment. If a woman is interested, a blood sample will be collected to determine if she meets the criteria for enrollment i.e. HBeAg positive or HBV

For peer review only - http://bmjopen.bmj.com/site/about/guidelines.xhtml

DNA >1.9 log 10 (>85 IU/mL). Women will be informed of the results at the next visit and be enrolled and started on TDF if all inclusion criteria are met (Table 1).

SMRU has conducted research for over two decades and the trust in antenatal services is high resulting in high follow up rates in ANC as well as in previous studies[36]. If a woman misses an appointment she will be contacted using her phone number if applicable or the healthcare workers will reach out to her community or family to find her.

Follow up

Medical and Obstetric Data

Vital signs (blood pressure, tympanic temperature, heart rate, respiratory rate, fetal heart rate); weight, height, age, symphysis fundal height, fetal gestational age and heartbeat; medical history review (including medications, vitamins and allergies), ethnicity, details of any past pregnancies, complications, place of delivery and outcome will be recorded; and physical examination completed (**Figure 2 and Table 2**).

Laboratory tests

A maternal whole blood sample obtained by venipuncture will be collected at study entry, monthly until delivery, at birth and at two post-partum visits (Figure 2). Maternal HBV DNA, alanine amniotransferase (ALT) and creatinine will be measured at all scheduled visits for the mother. Serum phosphate will be measured at baseline and at birth.

Monthly visits during pregnancy

From the time TDF commences, the pregnant women will return for a monthly study visit. Obstetric follow-up at each monthly visit will include vital signs, weight, symphysis fundal height measurement and fetal heart beat and concomitant medications will be recorded into the CRF. Fetal growth will be measured monthly by ultrasound (head circumference, abdominal circumference and femur length).

Adverse events (AE) will be reviewed, and reported as AE or serious AE (SAE) according to the problem. Compliance will be monitored by pill counting and a questionnaire on adherence.

For peer review only

Delivery

Pregnancy complications and mode of delivery will be recorded in the CRF. Anthropometric measurements of the infant will be done in the first hour of life and will include birth weight, length, head and arm circumference, by trained staff and according to standard operating procedures.

Vaccination: HBIG (if mother is HBeAg positive) and standard HBV vaccine (at birth and 2, 4 and 6 months of age) will be provided to the infants according to the Expanded Program on Immunization (EPI) [37-39].

At birth a 12 ml sample of cord blood, 6 ml into EDTA and 6 ml into a plain tube, will be collected for analysis of HBV DNA and tenofovir level and HBsAg reflecting the HBV status of the infant. Note this cord blood sample might not be collected if the woman is referred for caesarean section into the Public Hospital system.

A maternal serum sample will be used to measure ALT, creatinine, phosphorus, HBsAg (HBeAg only if the woman was HBeAg positive at baseline) and tenofovir level.

Post-partum follow-up

TDF will be continued until one month post-partum to decrease the risk of flares as the maternal immune system returns to baseline. All women will continue the study until the four month post-partum visit. At the 1 and 4 months post-partum visits ALT will be measured. If ALT concentrations are increased, but there is not a true flare, follow up will continue and repeat ALT testing will be done at six months. If the flare worsens or does not resolve, they will be placed on anti-HBV therapy and be reviewed by a physician.

All infants will be followed at 1, 2, 4 and 6 months. The infant will be screened for HBV at two months. At each visit infant anthropometry will be measured (body weight, length, head circumference) and feeding recorded. At six months neurodevelopment will be assessed. At month 2, 4 and 6 the infant will receive follow up HBV vaccinations.

For peer review only

For peer review only - http://bmjopen.bmj.com/site/about/guidelines.xhtml

Laboratory based stopping criteria

ALT: Monthly lab results will be compared to baseline. Grade 3 or higher elevations will be an indication to stop the drug Grade 3 or higher is >5x upper limit of normal (ULN) if baseline levels were below the ULN; or if baseline levels were >ULN and , then grade 3 or higher is >3.5x the baseline value. The upper limit of normal of ALT will be defined as (0-31 U/L) [40].

Creatinine: A creatinine value of more than 1.0 mg/dL will be an indication to stop the drug regardless of the baseline value [40].

Sample size

We calculated the sample size required to estimate the proportion of pregnant women expected to be undetectable at delivery with precision.

$$n = \frac{(1.96)^2 p(1-p)}{d^2}$$

There is no prior knowledge to predict the proportion of pregnant women expected to be undetectable at delivery, so we used the most conservative estimate. When this proportion is 50%, the required sample size is the largest. Based on a 95% confidence interval, a margin of error d = 8% and a conservative estimation of proportion p = 0.5, a sample size of n = 150 is required. We aim for n = 170 to allow for attrition.

Data analyses

HBV Viral kinetics

The Kaplan-Meier method will be used to estimate the median time from initiation of TDF to an HBV DNA <100 IU/ml including all women and then, based on the distribution of HBV DNA, stratified by high vs low HBV DNA groups. A Generalized Estimating Equation (GEE) model will be used to assess whether baseline HBV DNA and adherence to TDF are associated with the logarithmically transformed HBV DNA over time. A proportional hazard model will be constructed to assess the hazard ratio of reaching undetectable HBV DNA adjusting for the baseline HBV DNA concentration and adherence to TDF (# pills actually taken/# pills that should have been taken). For missing HBV DNA data, depending on the reasons for being missing, imputations using Multiple Imputations by Chained Equations (MICE) will be used. Sensitivity analyses will then be performed comparing analyses using complete and imputed data sets. The

For peer review only - http://bmjopen.bmj.com/site/about/guidelines.xhtml

For peer review only

proportion of women with undetectable HBV DNA at delivery will be reported with 95% confidence interval in all women and by high vs low HBV DNA groups. Median and interquartile range (IQR) of HBV DNA at baseline and each visit will be reported. Then HBV DNA will be logarithmically transformed and the median HBV DNA at baseline and at delivery will be compared using paired t-tests. Additional analyses may be conducted to further explore the data.

Adherence

The main analysis to determine adherence will be calculating percent adherence using pill counting data (# pills actually taken/# pills that should have been taken). As a confirmation of adherence, we will also measure TDF concentration in all women with detectable HBV DNA at delivery and in a matched subset of women who become undetectable for comparison (n=2x the number of women who do not become undetectable). This is anticipated to include 10-15 women who are HBV DNA detectable at delivery and matched to 20-30 women who are undetectable. This analytic sub-sample will be matched on age and initial DNA concentration or, if a matched pair analysis is not feasible because no "matching partners" exist, we will correct for these factors using multivariate regression methods.

The 'Adherence Starts with Knowledge' questionnaire ASK-12 is a tool to determine adherence and is derived from the ASK-20 questionnaire [41]. The questionnaire consists of 12 items related to drug adherence in three scales "forgetfulness", "treatment beliefs", and "behavior". Although previous studies have demonstrated the usefulness of ASK-12 for patients, this tool has not been validated in this study population [42, 43]. Local pilot testing has resulted in a culturally appropriate version of this questionnaire and personnel will be trained to use it. We will use pill counts (gold standard) as well as TDF blood concentration (confirmatory) to validate this tool. The results will be analysed by first comparing differences among the groups, one-way analysis of variance and Bonferroni's multiple comparison tests.

Hepatic Flare

Although TDF is thought to be safe and acceptable in pregnancy, after discontinuation it might lead to hepatitis flares. There are conflicting data about the proportion and severity of flares after discontinuing antiviral therapy [44-46]. There are no data in pregnant women regarding flares with longer duration of treatment and lower HBV DNA at delivery, nor is there information about predisposing characteristics to provide recommendations either before, or even after, flares start. We will determine the proportion of

For peer review only - http://bmjopen.bmj.com/site/about/guidelines.xhtml

woman who have a moderate (ALT>2.5x upper limit of normal) or severe (ALT>5x upper limit of normal) hepatitis flare after discontinuation of TDF [47]. We will estimate the impact of potential risk factors such as age, number of prior pregnancies, etc. for moderate or severe flares by including treatment duration and HBV DNA concentration at study start and at delivery into multivariable regression models.

Secondary analyses

We will screen for HBV infection by screening for HBsAg in infants at two months of age. If HBsAg is positive, presence of HBV DNA from the same blood sample will be confirmed. If MTCT of HBV exists, the time of TDF initiation, maternal baseline HBV DNA, and TDF adherence will be compared between mothers with MTCT and those without using t-test and Chi-square test/Fisher's exact test. Infant anthropometry will be reported at 1, 2, 4 and 6 months. We will compare the infant anthropometry to the same age infant in general population. Additional analyses such as correlation between TDF use time and infant anthropometry will be conducted based on the data we observe.

ETHICS AND DISSEMINATION

Ethical considerations

SMRU has worked with pregnant women for three decades and provided research results that have had significant local and international impact [30, 48]. Maternal and infant mortality have decreased significantly over the years SMRU has worked with the population. Women come to deliver with SMRU by choice. These marginalized populations and their ability to negotiate the health landscape may be limited by education level but this is recognized by local health staff who are from the same community and form part of the research team [30, 48]. All communication is conducted in the preferred language of the patient and additional efforts are made to assist non-literate women. We ensure that participation is voluntary and that it is clearly understood that participation in the study can be ceased at any time without effect to the care provided. Extra precautions to inform participants about discontinuation while under TDF are also part of the information sheets.

The Tak Community Advisory Board (T-CAB) reviewed the project for relevance, advice and acceptability in a meeting in relation to this study prior to submission of the protocol [49]. Pertinent responses from the T-CAB in relation to this study were positive but also included a request to include adolescent pregnant

For peer review only - http://bmjopen.bmj.com/site/about/guidelines.xhtml

women. Several T-CAB members discussed further the issue of consent and assent in relation to SMRU staff, pregnant women, study participants and guardians to further inform the study team on how this issue is best managed with the marginalized, mobile border population.

This study has been approved by the Ethics Committee of the Faculty of Tropical Medicine, Mahidol University (FTM ECF-019-06), Johns Hopkins University (IRB No: 00007432), Chiang Mai University (FAM-2559-04227), Oxford Tropical Research Ethics Committee (OxTREC Reference: 49-16) and by the local Tak Community Advisory Board (TCAB-02/REV/2016).

Informed consent

Trained counsellors will seek the mother's consent after the patient information sheet has been discussed. All queries from the mothers will be answered and those who agree will indicate consent by signing or providing thumbprints. All data collected will be treated confidentially and stored securely.

Patient and Public Involvement statement

The Tak Community Advisory Board (T-CAB) reviewed the project for relevance, advice and acceptability in a meeting in relation to this study prior to submission of the protocol. Pertinent responses from the T-CAB in relation to this study were positive but also included a request to include adolescent pregnant women. Several T-CAB members discussed further the issue of consent and assent in relation to SMRU staff, pregnant women, study participants and guardians to further inform the study team on how this issue is best managed with the marginalized, mobile border population.

For peer review only

List of abbreviations

AE	Adverse event
ANC	Antenatal Care
ASK	Adherence Starts with Knowledge
DNA	deoxyribonucleic acid
HBeAg	Hepatitis B e antigen
HBIG	Hepatitis B immunoglobulin
HBsAg	Hepatitis B surface antigen
HBV	Hepatitis B virus
LIC	Low-income country
MTCT	Mother to child transmission
SMRU	Shoklo Malaria Research Unit
TDF	Tenofovir Disoproxil Fumarate

For peer review only

Author Statements

KN, SE, CLT and RM designed the protocol; SE, KN and CLT obtained funding; RM, CLT, SE, FT, CA and MB obtained the Ethical approvals; RM and MB set up the clinical work; WW and CLL prepared laboratory testing; NG and YJ supported statistical considerations; PJ, VIC, FT, MvV, MJR and FN supported the design and clinical implementation of the study; all authors read and contributed to the present manuscript. All authors read and approved the final manuscript.

For peer review only - http://bmjopen.bmj.com/site/about/guidelines.xhtml

Data statement

After analyses of the data the data will be made available.

Funding

The work is supported by the following awards: Thrasher Research Fund [grant number: TRF13096] to Johns Hopkins University; the Wellcome-Trust Major Overseas Program in Southeast Asia [grant number: 106698/Z/14/Z] to Mahidol University Oxford Tropical Medicine Research Programme which directly supports PJ, CLL, FN, RM from Shoklo Malaria Research Unit; and Chiang Mai University Thailand supporting MB, FT and CA.

Competing interests

The authors declare that they have no competing interests

Acknowledgements

We would kindly like to acknowledge all contributions of SMRU staff and doctors for their feedback in finalizing the research proposal. Also the data management team for contributing to the Case Report Form development and our quality control team, Moo Kho Paw and Rattanaporn Raksapraidee, and clinical trials support group, for their efforts. Myo Chit Min helped to design the map.

References

1. WHO. Guidelines for the prevention, care and treatment of persons with chronic hepatitis B infection. Geneva: World Health Organization; 2015. Report No.: NLM classification: WC 536.”. . Available at: https://www.who.int/hepatitis/publications/hepatitis-b-guidelines/en/. Accessed 19 December 2019.

2. Thomas DL. Global Elimination of Chronic Hepatitis. N Engl J Med **2019**; 380(21): 2041-50.

3. Bloom DE, Khoury A, Subbaraman R. The promise and peril of universal health care. Science (New York, NY) **2018**; 361(6404).

4. Banks T, Kang J, Watts I, et al. High hepatitis B seroprevalence and risk factors for infection in pregnant women on the Thailand-Myanmar Border. J Infect Dev Ctries **2016**; 10(4): 384-8.

For peer review only - http://bmjopen.bmj.com/site/about/guidelines.xhtml

5. Bierhoff M, Angkurawaranon C, Myat Min A, et al. Maternal Hepatitis B Infection Burden, Comorbidity and Pregnancy Outcome in a Low-Income Population on the Myanmar-Thailand Border: A Retrospective Cohort Study. J Pregnancy **2019**; 2019: 8435019.

6. Pan CQ, Duan Z, Dai E, et al. Tenofovir to Prevent Hepatitis B Transmission in Mothers with High Viral Load. N Engl J Med **2016**; 374(24): 2324-34.

7. Thio CL, Guo N, Xie C, Nelson KE, Ehrhardt S. Global elimination of mother-to-child transmission of hepatitis B: revisiting the current strategy. Lancet Infect Dis **2015**; 15(8): 981-5.

8. Lattof SR, Tuncalp O, Moran AC, et al. Developing measures for WHO recommendations on antenatal care for a positive pregnancy experience: a conceptual framework and scoping review. BMJ open **2019**; 9(4): e024130.

9. Li XM, Yang YB, Hou HY, et al. Interruption of HBV intrauterine transmission: a clinical study. World J Gastroenterol **2003**; 9(7): 1501-3.

10. Beigi R, Noguchi L, Parsons T, et al. Pharmacokinetics and placental transfer of single-dose tenofovir 1% vaginal gel in term pregnancy. J Infect Dis **2011**; 204(10): 1527-31.

11. Thilakanathan C, Wark G, Maley M, et al. Mother-to-child transmission of hepatitis B: Examining viral cut-offs, maternal HBsAg serology and infant testing. Liver international : official journal of the International Association for the Study of the Liver **2018**; 38(7): 1212-9.

12. Wen WH, Chang MH, Zhao LL, et al. Mother-to-infant transmission of hepatitis B virus infection: significance of maternal viral load and strategies for intervention. J Hepatol **2013**; 59(1): 24-30.

13. Lu Y, Zhu FC, Liu JX, et al. The maternal viral threshold for antiviral prophylaxis of perinatal hepatitis B virus transmission in settings with limited resources: A large prospective cohort study in China. Vaccine **2017**; 35(48 Pt B): 6627-33.

14. Jourdain G, Ngo-Giang-Huong N, Harrison L, et al. Tenofovir versus Placebo to Prevent Perinatal Transmission of Hepatitis B. New England Journal of Medicine **2018**; 378(10): 911-23.

15. Lee YS, Lee HS, Kim JH, et al. Role of tenofovir disoproxil fumarate in prevention of perinatal transmission of hepatitis B virus from mother to child: a systematic review and meta-analysis. The Korean journal of internal medicine **2019**.

For peer review only

For peer review only - http://bmjopen.bmj.com/site/about/guidelines.xhtml

16. Gao F, Zhang WT, Lin YY, Wang WM, Xu N, Bai GQ. Early Start Of Tenofovir Treatment Achieves Better Viral Suppression In Pregnant Women With A High HBV Viral Load: A Real-World Prospective Study. Infection and drug resistance **2019**; 12: 3475-84.

17. Thio CL, Guo N, Xie C, Nelson KE, Ehrhardt S. Global elimination of mother-to-child transmission of hepatitis B: revisiting the current strategy. The Lancet Infectious Diseases **2015**; 15(8): 981-5.

18. Colbers AP, Hawkins DA, Gingelmaier A, et al. The pharmacokinetics, safety and efficacy of tenofovir and emtricitabine in HIV-1-infected pregnant women. AIDS (London, England) **2013**; 27(5): 739-48.

19. Pyra M, Anderson PL, Hendrix CW, et al. Tenofovir and tenofovir-diphosphate concentrations during pregnancy among HIV-uninfected women using oral preexposure prophylaxis. AIDS (London, England) **2018**; 32(13): 1891-8.

20. Bierhoff M, Pinyopornpanish K, Pinyopornpanish K, et al. Retrospective Review of Documentation Practices of Hepatitis B Immunoglobulin, Birth Dose, and Vaccination at the Hospital of Birth, in Thai Nationals and Migrants in Northern Thailand. Open forum infectious diseases **2019**; 6(12): ofz518.

21. Best BM, Burchett S, Li H, et al. Pharmacokinetics of tenofovir during pregnancy and postpartum. HIV Med **2015**; 16(8): 502-11.

22. Benaboud S, Hirt D, Launay O, et al. Pregnancy-related effects on tenofovir pharmacokinetics: a population study with 186 women. Antimicrob Agents Chemother **2012**; 56(2): 857-62.

23. Bierhoff M, Smolders EJ, Tarning J, et al. Pharmacokinetics of oral tenofovir disoproxil fumarate in pregnancy and lactation: a systematic review. Antiviral therapy **2019**; 24(7): 529-40.

24. Cressey TR, Avihingsanon A, Halue G, et al. Plasma and Intracellular Pharmacokinetics of Tenofovir Disoproxil Fumarate 300 mg Every 48 Hours vs 150 mg Once Daily in HIV-Infected Adults With Moderate Renal Function Impairment. Clin Infect Dis **2015**; 61(4): 633-9.

25. Siberry GK, Jacobson DL, Kalkwarf HJ, et al. Lower Newborn Bone Mineral Content Associated With Maternal Use of Tenofovir Disoproxil Fumarate During Pregnancy. Clin Infect Dis **2015**; 61(6): 996-1003.

26. Van Dyke RB, Chadwick EG, Hazra R, Williams PL, Seage GR, 3rd. The PHACS SMARTT Study: Assessment of the Safety of In Utero Exposure to Antiretroviral Drugs. Front Immunol **2016**; 7: 199.

For peer review only - http://bmjopen.bmj.com/site/about/guidelines.xhtml

27. Yankam BM, Anye CS, Nkfusai NC, Shirinde J, Cumber SN. Knowledge and practice of pregnant women and health care workers on hepatitis B prevention in the Limbe and Muyuka health districts of the south west region of Cameroon. The Pan African medical journal **2019**; 33: 310.

28. Durante-Mangoni E, Vitrone M, Parrella A, et al. Efficacy and safety of tenofovir, entecavir, and telbivudine for chronic hepatitis B in heart transplant recipients. Transpl Infect Dis **2016**; 18(3): 319-25.

29. Suk-Fong Lok A. Hepatitis B Treatment: What We Know Now and What Remains to Be Researched. Hepatology communications **2019**; 3(1): 8-19.

30. Gilder ME, Moo P, Hashmi A, et al. "I can't read and don't understand": Health literacy and health messaging about folic acid for neural tube defect prevention in a migrant population on the Myanmar-Thailand border. PLoS One **2019**; 14(6): e0218138.

31. Manoyos V TA, Srithanaviboonchai K, Yangyuenkul S, Grimes RM. Sexual risk-behaviors for HIV infections among young cross-border migrant workers living in urban Chiang Mai, Thailand. Journal of Health Research **2016**; 30(5): 347-53.

32. International, Organization, for, Imigration. IOM Thailand National Strategy 2017-2020. Available at: https://thailand.iom.int/iom-thailand-national-strategy-2017-2020. Accessed June 2019.

33. Meyer SR, Decker MR, Tol WA, Abshir N, Mar AA, Robinson WC. Workplace and security stressors and mental health among migrant workers on the Thailand-Myanmar border. Social psychiatry and psychiatric epidemiology **2016**; 51(5): 713-23.

34. Culhane-Pera KA, Sriphetcharawut S, Thawsirichuchai R, Yangyuenkun W, Kunstadter P. Afraid of Delivering at the Hospital or Afraid of Delivering at Home: A Qualitative Study of Thai Hmong Families' Decision-Making About Maternity Services. Matern Child Health J **2015**; 19(11): 2384-92.

35. Suphanchaimat R, Putthasri W, Prakongsai P, Tangcharoensathien V. Evolution and complexity of government policies to protect the health of undocumented/illegal migrants in Thailand - the unsolved challenges. Risk Manag Healthc Policy **2017**; 10: 49-62.

36. Fellmeth G, Plugge EH, Carrara V, et al. Migrant perinatal depression study: a prospective cohort study of perinatal depression on the Thai-Myanmar border. BMJ open **2018**; 8(1): e017129.

37. WHO. Expanded Program on Immunization Factsheet Myanmar. Available at: http://www.searo.who.int/immunization/data/myanmar.pdf. Accessed December 2018.

For peer review only - http://bmjopen.bmj.com/site/about/guidelines.xhtml

For peer review only

38. Cutts FT, Claquin P, Danovaro-Holliday MC, Rhoda DA. Monitoring vaccination coverage: Defining the role of surveys. Vaccine **2016**; 34(35): 4103-9.

39. Schalm SW, Mazel JA, de Gast GC, et al. Prevention of hepatitis B infection in newborns through mass screening and delayed vaccination of all infants of mothers with hepatitis B surface antigen. Pediatrics **1989**; 83(6): 1041-8.

40. Abbassi-Ghanavati M, Greer LG, Cunningham FG. Pregnancy and laboratory studies: a reference table for clinicians. Obstet Gynecol **2009**; 114(6): 1326-31.

41. Matza LS, Park J, Coyne KS, Skinner EP, Malley KG, Wolever RQ. Derivation and validation of the ASK-12 adherence barrier survey. The Annals of pharmacotherapy **2009**; 43(10): 1621-30.

42. Sasaki J, Kawayama T, Yoshida M, et al. Adherence barriers to inhaled medicines in Japanese older patients with asthma evaluated using the "Adherence Starts with Knowledge 20" (ASK-20) questionnaire. The Journal of asthma : official journal of the Association for the Care of Asthma **2019**; 56(6): 632-41.

43. Kimura Y, Koya T, Hasegawa T, et al. Characterization of low adherence population in asthma patients from Japan using Adherence Starts with Knowledge-12. Allergology international : official journal of the Japanese Society of Allergology **2020**; 69(1): 61-5.

44. Nguyen V, Tan PK, Greenup AJ, et al. Anti-viral therapy for prevention of perinatal HBV transmission: extending therapy beyond birth does not protect against post-partum flare. Aliment Pharmacol Ther **2014**; 39(10): 1225-34.

45. Xu WM, Cui YT, Wang L, et al. Lamivudine in late pregnancy to prevent perinatal transmission of hepatitis B virus infection: a multicentre, randomized, double-blind, placebo-controlled study. Journal of viral hepatitis **2009**; 16(2): 94-103.

46. Liem KS, Gehring AJ, Feld JJ, Janssen HLA. Challenges with stopping long-term nucleos(t)ide analogue therapy in patients with chronic hepatitis B. Gastroenterology **2019**.

47. Wang L, Kourtis AP, Ellington S, Legardy-Williams J, Bulterys M. Safety of tenofovir during pregnancy for the mother and fetus: a systematic review. Clin Infect Dis **2013**; 57(12): 1773-81.

48. Parker DM, Carrara VI, Pukrittayakamee S, McGready R, Nosten FH. Malaria ecology along the Thailand-Myanmar border. Malar J **2015**; 14: 388.

For peer review only

49. Pratt B, Lwin KM, Zion D, Nosten F, Loff B, Cheah PY. Exploitation and community engagement: can community advisory boards successfully assume a role minimising exploitation in international research? Dev World Bioeth **2015**; 15(1): 18-26.

For peer review only

Figure 1. Map of the central office of SMRU in Mae Sot, and the study sites Wang Pha (WPA), Mawker Thai (MKT) and Maela (MLA) on the Myanmar-Thailand border where the study will be conducted.

For peer review only

For peer review only - http://bmjopen.bmj.com/site/about/guidelines.xhtml

Figure 2. Schematic reproduction of the study design

ALT, Alanine Aminotransferase; Cr, Creatinine; DNA, deoxyribonucleic acid; EGA, estimated gestational age; HBeAg, Hepatitis B envelope antigen; HBIG, Hepatitis B immunoglobulins; HBsAg, Hepatitis B surface antigen; HBV, Hepatitis B virus; TDF, Tenofovir Disoproxil Fumarate

For peer review only

For peer review only - http://bmjopen.bmj.com/site/about/guidelines.xhtml

Table 1. Inclusion and exclusion criteria

Inclusion criteria	Exclusion criteria
Willing and able to give informed consent for participation in the study	Negative qualitative HBV DNA if HBeAg negative
Hepatitis B infected (HBsAg confirmed positive and qualitative HBV DNA positive in HBeAg negative mothers)	HIV positive or on immunosuppressive therapy for other illnesses
Female, 16-49 years	Elevated creatinine (>1 mg/dL)
EGA[1] 12-20 weeks at start TDF[2]	Abnormal serum phosphate (<2.4 and >4.5 mg/dL)
Willing to take TDF[2] daily during pregnancy	History of kidney disease
Plans to deliver at SMRU[3]	History of pregnancy complications

[1]Estimated Gestational Age, [2]Tenofovir Disoproxil Fumarate, [3]Shoklo Malaria Research Unit

Table 2. Overview of measurements and interventions per visit

**Procedures**	**Visits timing**						
	**Start**	**Monthly visits**	**Delivery**	**1 month PP**	**2 months PP**	**4 months PP**	**6 months PP**
		**On TDF**	**On TDF**	**Stop TDF**		**End mother**	**End infant**
Eligibility checklist	X						
Informed consent	X						
Ultrasound	X	X	X				
Demographics	X						
Medical history	X						
Physical examination	X	X	X	X		X	
**Laboratory tests**							
HBsAg/HBeAg	X						
HBV DNA	X	X	X	X			
Creatinine	X	X	X	X			
Serum Phosphate	X		X				
ALT	X	X	X	X		X[1]	
TDF Concentration			X[2]				
Cord HBsAg/HBV DNA			X[2]				

For peer review only - http://bmjopen.bmj.com/site/about/guidelines.xhtml
For peer review only

Infant HBsAg					X[4]		
Pill count		X	X	X			
Adverse event assessments		X	X	X			
Infant anthropometry			X	X	X	X	X
Infant neurodevelopment							X
Adherence survey		X	X	X			
Infant HB vaccination			X[3]		X	X	X

[1] Follow-up will continue if suspected or confirmed flare, [2] Cord blood sample, [3]If mother HBeAg positive also HBIG , [4] Venepuncture sample blood and only for HBsAg

For peer review only

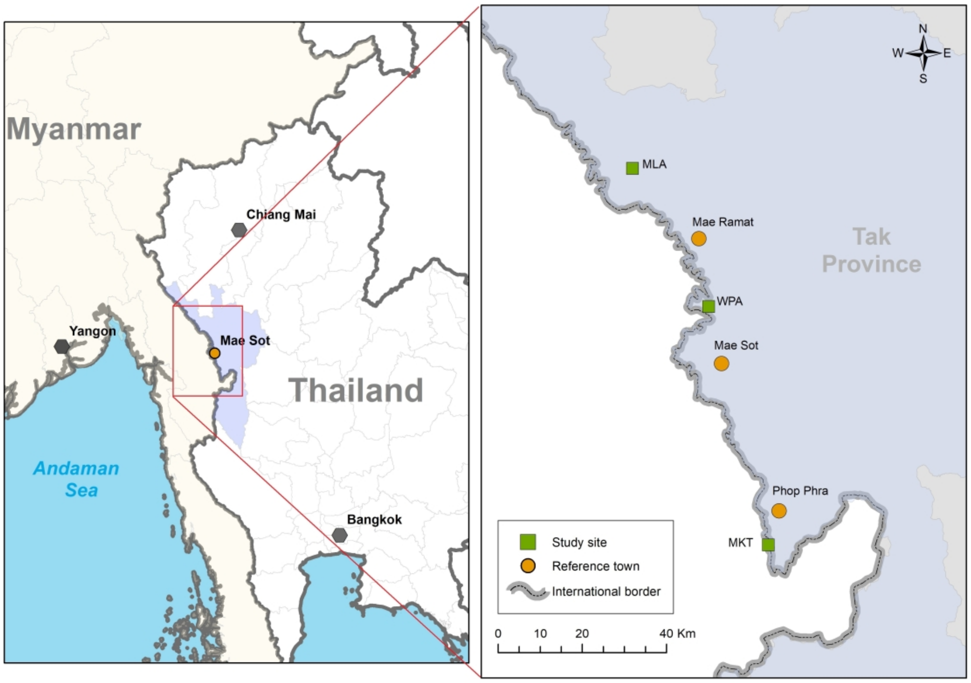

Figure 1. Map of the central office of SMRU in Mae Sot, and the study sites Wang Pha (WPA), Mawker Thai (MKT) and Maela (MLA) on the Myanmar-Thailand border where the study will be conducted.

296x210mm (400 x 400 DPI)

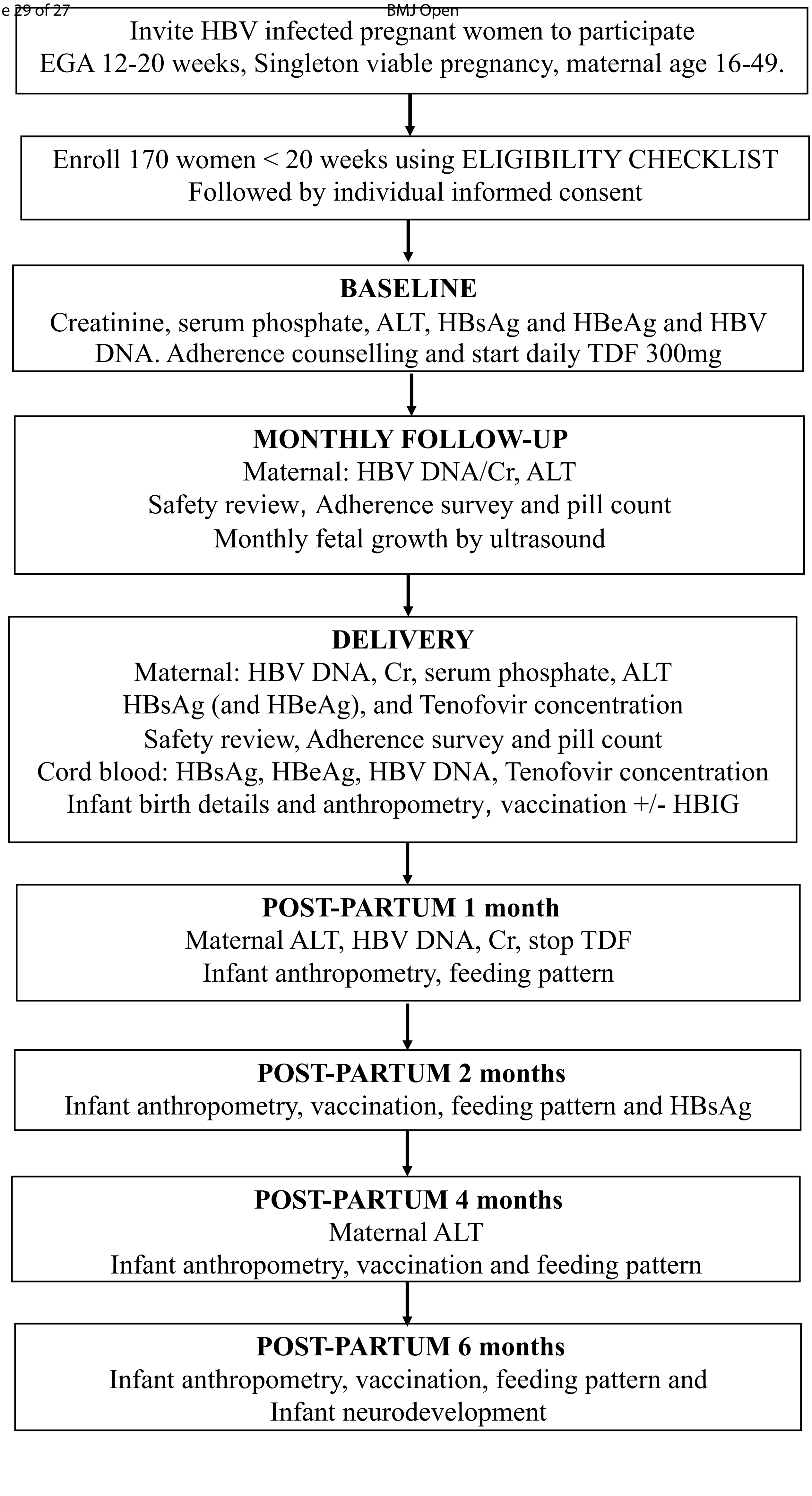
Invite HBV infected pregnant women to participate
EGA 12-20 weeks, Singleton viable pregnancy, maternal age 16-49.
Enroll 170 women < 20 weeks using ELIGIBILITY CHECKLIST
Followed by individual informed consent
BASELINE
Creatinine, serum phosphate, ALT, HBsAg and HBeAg and HBV DNA. Adherence counselling and start daily TDF 300mg
MONTHLY FOLLOW-UP
Maternal: HBV DNA/Cr, ALT
Safety review, Adherence survey and pill count
Monthly fetal growth by ultrasound
DELIVERY
Maternal: HBV DNA, Cr, serum phosphate, ALT
HBsAg (and HBeAg), and Tenofovir concentration
Safety review, Adherence survey and pill count
Cord blood: HBsAg, HBeAg, HBV DNA, Tenofovir concentration
Infant birth details and anthropometry, vaccination +/- HBIG
POST-PARTUM 1 month
Maternal ALT, HBV DNA, Cr, stop TDF
Infant anthropometry, feeding pattern
POST-PARTUM 2 months
Infant anthropometry, vaccination, feeding pattern and HBsAg
POST-PARTUM 4 months
Maternal ALT
Infant anthropometry, vaccination and feeding pattern
POST-PARTUM 6 months
Infant anthropometry, vaccination, feeding pattern and
Infant neurodevelopment

BMJ Open

Prevention of mother-to-child transmission of hepatitis B virus: protocol for a one arm, open label intervention study to estimate the optimal timing of tenofovir in pregnancy

Journal:	*BMJ Open*
Manuscript ID	bmjopen-2020-038123.R2
Article Type:	Protocol
Date Submitted by the Author:	29-Jun-2020
Complete List of Authors:	Bierhoff, Marieke; Shoklo Malaria Research Unit; Amsterdam UMC, Internal Medicine and Tropical Diseases<br>Nelson, Kenrad; Johns Hopkins University, Department of Epidemiology<br>Guo, Nan; Johns Hopkins University, Department of Anesthesiology<br>Jia, Yuanxi ; Johns Hopkins University, Department of Epidemiology<br>Angkurawaranon, Chaisiri ; Chiang Mai University, Department of Family Medicine<br>Jittamala, Podjanee ; Mahidol Oxford Tropical Medicine Research Unit; Mahidol University Faculty of Medicine Ramathibodi Hospital, Department of Tropical Hygiene<br>Carrara, Verena; Shoklo Malaria Research Unit, MCH<br>Watthanaworawit, Wanitda ; Shoklo Malaria Research Unit<br>Ling, Clare; Shoklo Malaria Research Unit; Oxford University, Centre for Tropical Medicine and Global Health<br>Tongprasert, Fuanglada; Chiang Mai University, Department of Obstetrics and Gynecology<br>van Vugt, Michele; Amsterdam UMC, Internal Medicine and Tropical Diseases<br>Rijken, Marcus; Utrecht University, Department of Obstetrics and Gynecology<br>Nosten, Francois; Oxford University, Centre for Tropical Medicine and Global Health; Oxford University, Centre for Tropical Medicine and Global Health<br>McGready, Rose; Shoklo Malaria Research Unit, Faculty of Tropical Medicine, Mahidol University; Oxford University, Centre for Tropical Medicine and Global Health<br>Ehrhardt, Stephan ; Johns Hopkins University, Department of Epidemiology<br>Thio, Chloe; Johns Hopkins University School of Medicine,
**Primary Subject Heading**:	Infectious diseases
Secondary Subject Heading:	Global health, Obstetrics and gynaecology, Public health, Reproductive medicine
Keywords:	Public health < INFECTIOUS DISEASES, Tropical medicine < INFECTIOUS DISEASES, Fetal medicine < OBSTETRICS, Maternal medicine < OBSTETRICS, THERAPEUTICS, VIROLOGY

For peer review only - http://bmjopen.bmj.com/site/about/guidelines.xhtml

SCHOLARONE™
Manuscripts

I, the Submitting Author has the right to grant and does grant on behalf of all authors of the Work (as defined in the below author licence), an exclusive licence and/or a non-exclusive licence for contributions from authors who are: i) UK Crown employees; ii) where BMJ has agreed a CC-BY licence shall apply, and/or iii) in accordance with the terms applicable for US Federal Government officers or employees acting as part of their official duties; on a worldwide, perpetual, irrevocable, royalty-free basis to BMJ Publishing Group Ltd ("BMJ") its licensees and where the relevant Journal is co-owned by BMJ to the co-owners of the Journal, to publish the Work in this journal and any other BMJ products and to exploit all rights, as set out in our licence.

The Submitting Author accepts and understands that any supply made under these terms is made by BMJ to the Submitting Author unless you are acting as an employee on behalf of your employer or a postgraduate student of an affiliated institution which is paying any applicable article publishing charge ("APC") for Open Access articles. Where the Submitting Author wishes to make the Work available on an Open Access basis (and intends to pay the relevant APC), the terms of reuse of such Open Access shall be governed by a Creative Commons licence – details of these licences and which Creative Commons licence will apply to this Work are set out in our licence referred to above.

Other than as permitted in any relevant BMJ Author's Self Archiving Policies, I confirm this Work has not been accepted for publication elsewhere, is not being considered for publication elsewhere and does not duplicate material already published. I confirm all authors consent to publication of this Work and authorise the granting of this licence.

For peer review only

Prevention of mother-to-child transmission of hepatitis B virus: protocol for a one arm, open label intervention study to estimate the optimal timing of tenofovir in pregnancy

Marieke Bierhoff[1, 2], Kenrad E. Nelson[3], Nan Guo[4], Yuanxi Jia[3], Chaisiri Angkurawaranon[5], Podjanee Jittamala[6,7], Verena I Carrara[1], Wanitda Watthanaworawit[1], Clare L. Ling[1,8], Fuanglada Tongprasert[9], Michele van Vugt[2], Marcus J. Rijken[10], Francois Nosten[1,8], Rose McGready[1,8], Stephan Ehrhardt[3*], Chloe L. Thio[11*]

[1]Shoklo Malaria Research Unit, Mahidol-Oxford Tropical Medicine Research Unit, Faculty of Tropical Medicine, Mahidol University, Mae Sot, Thailand

[2]Department of Internal Medicine and Tropical Diseases, Academic Medical Center Amsterdam, the Netherlands

[3] Department of Epidemiology, Johns Hopkins Bloomberg School of Public Health, Baltimore, MD, USA

[4] Department of Anesthesiology, Perioperative and Pain Medicine, Stanford University, Stanford, CA, USA

[5] Department of Family Medicine, Faculty of Medicine, Chiang Mai University

[6]Mahidol-Oxford Tropical Medicine Research Unit, Faculty of Tropical Medicine, Mahidol University, Bangkok, Thailand

[7] Department of Tropical Hygiene, Faculty of Tropical Medicine, Mahidol University, Bangkok, Thailand

[8] Centre for Tropical Medicine and Global Health, Nuffield Department of Medicine, University of Oxford, Oxford, UK

[9] Department of Obstetrics and Gynecology, Faculty of Medicine, Chiang Mai University

[10]Department of Obstetrics and Gynecology, Utrecht University Medical Centre, Utrecht, Netherlands

[11] Department of Medicine, Johns Hopkins University School of Medicine, Baltimore, MD, USA

For peer review only

For peer review only - http://bmjopen.bmj.com/site/about/guidelines.xhtml

Corresponding author: Stephan Ehrhardt, Department of Epidemiology and Internal Medicine, Johns Hopkins University, School of Medicine 855 N Wolfe Street, 5th floor, Baltimore, MD 21205, e-mail: sehrhar6@jhu.edu

* Contributed equally

Word count: 3,645

Keywords: HBV, Antiviral therapy, maternal health

For peer review only

For peer review only - http://bmjopen.bmj.com/site/about/guidelines.xhtml

ABSTRACT

Introduction: Hepatitis B Virus (HBV) remains a public health threat and the main route of transmission is from mother to child (MTCT). Tenofovir disoproxil fumarate (TDF) treatment can reduce MTCT of HBV although the optimal timing to attain undetectable HBV DNA concentrations at delivery is unknown. This protocol describes the procedures following early initiation of maternal TDF prior to 20 weeks gestation to determine efficacy, safety and feasibility of this approach in a limited-resource setting.

Methods and analyses: One hundred and seventy pregnant women from the Thailand-Myanmar border between 12 to <20 weeks gestational age will be enrolled into a one arm, open label, TDF treatment study with cessation of TDF one month after delivery. Sampling occurs monthly prenatal, at birth and at 1, 2, 4 and 6 months post-partum. Measurement of tenofovir concentrations in maternal and cord plasma is anticipated in 10-15 women who have detectable HBV DNA at delivery and matched to 20-30 women with no detectable HBV DNA. Infant HBsAg status will be determined at two months of age and HBV DNA confirmed in HBsAg positive cases. Adverse events including risk of flare and adherence, based on pill count and questionnaire, will be monitored. Infants will receive HBV vaccinations at birth, 2, 4 and 6 months and hepatitis B immunoglobulin at birth if the mother is hepatitis B e antigen positive. Infant growth and neurodevelopment at six months will be compared to established local norms.

Ethics and dissemination: This study has ethical approval by the Ethics Committee of the Faculty of Tropical Medicine, Mahidol University (FTM ECF-019-06), Johns Hopkins University (IRB No: 00007432), Chiang Mai University (FAM-2559-04227), Oxford Tropical Research Ethics Committee (OxTREC Reference: 49-16) and by the local Tak Community Advisory Board (TCAB-02/REV/2016). The article will be published as an open access publication. ClinicalTrials.gov Identifier: NCT02995005

For peer review only - http://bmjopen.bmj.com/site/about/guidelines.xhtml

ARTICLE SUMMARY

Strengths and limitations of this study

1. This prospective cohort study will inform the optimal time to initiate tenofovir disoproxil fumarate (TDF) treatment in pregnant women to reduce Hepatitis B virus (HBV) DNA to undetectable concentrations (defined as <100 IU/ml) prior to birth, which is a strength since this is currently unknown.
2. Strategies for prevention of mother to child transmission (MTCT) of human immunodeficiency virus (HIV) are not immediately transferable to HBV so another strength is that this study investigates the feasibility of implementing the recommended treatment for HBV in pregnant women in a limited-resource setting.
3. It is important to identify challenges to adherence and safety in a context of low literacy where two thirds of pregnant women have limited education to ensure that potential implementation of this strategy is feasible
4. This study investigates TDF usage during pregnancy for prevention of MTCT as a potential alternative to hepatitis B immunoglobulin (HBIG), which remains too expensive and difficult to obtain in most limited-resource settings, and will inform future studies that forgo HBIG.
5. This study investigates TDF in addition to HBIG and vaccination in infants born to hepatitis B e antigen positive mothers as this is the most comprehensive way to prevent MTCT, but such a design does not provide information on the proportion of MTCT in women with an undetectable HBV DNA without HBIG.

For peer review only - http://bmjopen.bmj.com/site/about/guidelines.xhtml

INTRODUCTION

Background and rationale

In 2016 it was estimated that only 1 in 10 of the 257 million people living with hepatitis B virus (HBV) were aware of their infection and of those, 1 in 7 were on treatment, which is less than 2% of the HBV infections globally [1]. The current sustainable development goals aim to reduce the prevalence of HBV by 90% by 2030 [2]. An essential route to reach this goal is prevention of mother to child transmission (MTCT) of HBV, the most common source of infection. Pregnancy provides a window of opportunity in the life of an adult to advocate for a screening intervention, raising awareness of HBV infection, potentially resulting in positive impacts on prevalence. Unfortunately both screening and prevention efforts are impeded by lack of access to universal health care and essential health services, a problem relevant to half of the world's population [3].

Maternal HBV infection is an important communicable disease on the Myanmar-Thailand border [4] and in other parts of Asia, the western Pacific and African Region, where more than 6% of adults are infected [1]. In antenatal clinics at the Shoklo Malaria Research Unit (SMRU) clinics, the hepatitis B surface antigen (HBsAg) prevalence is 6.4-8.3% with a hepatitis B e antigen (HBeAg) prevalence of 32.7% in those positive for HBsAg [4, 5]. Mothers who are HBeAg positive are at the highest risk of transmitting HBV to their infants, which is the basis for specifically targeting this group for neonatal hepatitis B immunoglobulins (HBIG) within 72 hours of life. In infants born to HBeAg positive mothers and provided HBIG and HBV vaccine, prevention of MTCT fails in an estimated 8–32% of cases [6]. Overall, there are two reasons to find alternatives to HBIG: i) failure to prevent MTCT in infants born in HBeAg positive women, and ii) for the 50% of the world's population without universal health care who lack access to HBIG as it remains a specialized product that is costly, requires a cold chain and has a restrictive prescription window to be effective [7].

The usage of antivirals for HIV in limited-resource settings has shown remarkable scale up and success and is a vital component of antenatal care programs [8]. Antivirals that are active against HBV like tenofovir disoproxil fumarate (TDF) may reduce the risk of MTCT by reducing the HBV DNA across gestation to a minimum at the time of delivery when MTCT typically occurs and might be a more feasible option than HBIG in a limited-resource setting [9]. The optimal gestational age to commence TDF is critical to know to

For peer review only - http://bmjopen.bmj.com/site/about/guidelines.xhtml

ensure HBV DNA is as low as possible prior to birth [10]. The precise threshold of HBV DNA, in the absence of HBIG that prevents MTCT is also still unknown. Initially, a HBV DNA of ≥6 log10 IU/ml was associated with a higher risk of MTCT, suggesting women in this category were the most important to target for antiviral therapy [11-13]. Although a maternal HBV DNA <6 log10 IU/ml is thought to be lower risk for MTCT the studies reporting this provided the infants with HBIG and vaccination. Although provision of TDF 300 mg daily in the third trimester of pregnancy significantly reduced perinatal transmission (OR, 0.10; 95% CI, 0.01 to 0.77; p=0.03) [6, 14-16], a mean of 8.6 ± 0.5 weeks of TDF prior to delivery resulted in a mean HBV DNA at delivery of 4 log 10 IU/ml with 32% of mothers having HBV DNA >5.3 log 10 (or 200,000) IU/ml [6]. This amount of HBV DNA is too high to forego HBIG, and if provided, may still not be sufficient to prevent infection. In the same study HBV DNA declined another log (median 3.52 log10 IU/ml) in the four weeks post-partum that TDF was provided. Gao *et al.,* showed that starting TDF treatment at an estimated gestational age of 24–27 weeks may achieve better suppression compared to starting in third trimester [16].

Existing data from trials involving non-pregnant patients are not informative due to differences in age, stage of HBV, frequency of HBV DNA testing, and immune response and pharmacokinetics of TDF in pregnant women compared to non-pregnant patients [17-20]. Pharmacokinetic studies have established that the area under the curve (AUC) and maximum drug concentration (C_{max}) of TDF are decreased in the second and third trimester of pregnancy, and clearance is increased[18, 21-24]. In a systematic review of TDF pharmacokinetics in pregnancy, none of the manuscripts reported reaching the TDF C_{max} of 300 ng/ml the efficacy threshold determined in non-pregnant HBV infected women [23]. These data suggest that TDF dosage and treatment initiation that are accurate for HIV and non-pregnant HBV infected patients may not be accurate for pregnant HBV infected women.

While some of the safety issues have been resolved with the use of TDF for prevention of MTCT of HIV, gaps remain regarding HBV [25, 26]. The main barriers to implementation of TDF are a 1) lack of knowledge by the women of hepatitis B, of their infection status, and of prevention strategies [27], 2) poor adherence to the prophylactic drug regimen [9], and 3) potential for hepatitis flares after post-partum drug discontinuation [28]. All of these need to be evaluated and addressed in limited-resource settings where HBV is highly endemic [29].

For peer review only - http://bmjopen.bmj.com/site/about/guidelines.xhtml

The aim of this prospective interventional study is to determine the appropriate time in pregnancy to initiate TDF treatment of HBV in order to reach HBV DNA <100 IU/ml at delivery. Knowing this will allow future studies where TDF can be initiated at the appropriate time so that HBIG may not be needed to prevent MTCT of HBV. The viral kinetics of HBV DNA in pregnant women receiving daily oral TDF commenced before 20 weeks gestation will be measured monthly. Infants will receive HBIG and birth-dose monovalent HBV vaccine. MTCT will be assessed at two months post-partum. While the study population is pregnant women attending antenatal and birth services on the Myanmar-Thailand border, the study has worldwide application to women who are pregnant and HBV positive. Moreover, we will study safety, feasibility and potential barriers to implementation of TDF in routine antenatal care (ANC) in a limited-resource setting.

Objectives

The primary objective is to estimate the time to HBV DNA suppression <2 log10 IU/ml (or <100 IU/ml) in HBV DNA positive women who start TDF late in the first or early in the second trimester.

Secondary objectives include: estimation of the proportion of women with HBV DNA <2 log10 IU/ml (or <100 IU/ml) at delivery; the concentration of TDF in maternal and cord blood at birth in women who have detectable HBV DNA and a subset of women with undetectable HBV DNA; to monitor the safety of TDF on the Myanmar-Thailand border in pregnant women with low health literacy rates [30] and address potential barriers to implementing TDF in early pregnancy to prevent MTCT of HBV; to determine the rate of hepatic flares post-partum after cessation of TDF; to estimate the proportion of cases of vertical transmission in infants of two months of age; to assess fetal growth by monthly ultrasound, and infant growth at 1, 2 ,4 and 6 months and neurodevelopment at six months.

Study design

This is a prospective, one arm, open label, intervention study. Women who enroll in the study will be provided with a daily 300 mg dose of oral TDF starting at enrollment until one month after delivery (ClinicalTrials.gov Identifier: NCT02995005). Follow-up will continue at 1, 2, 4 and 6 months post-partum. A small sub-group of women are expected to require ongoing treatment for HBV flares and they will be followed for a further 6-9 months and provided drug therapy if required.

For peer review only - http://bmjopen.bmj.com/site/about/guidelines.xhtml

METHODS AND ANALYSIS

Setting

The study will be set in the clinics of Shoklo Malaria Research Unit (SMRU) on the border of Thailand and Myanmar (Figure 1). Since the 1990s the migrant influx from Myanmar to Thailand has increased for economic and political reasons, and to date there are approximately 3.5 million migrants in Thailand [31]. Over 25% of the migrant workers have no work permits and no legal status in Thailand [32]. Migrants are highly mobile, and the majority do not have access to basic health care which, combined with scarce services in Myanmar, put them at increased risk when seeking health services. In rural areas, labour migrants may be paid daily wages below Thailand's legal minimum wage reducing health-seeking behaviors which incur cost [33].

SMRU has provided humanitarian health care for marginalized populations for 30 years. One of the original objectives was to reduce malaria related maternal mortality from *Plasmodium falciparum* with screening embedded into routine maternal and child health care. The study will invite participants from three clinic sites (Mawker Thai, Wang Pha and Maela) serving marginalized populations (Figure 1). The long experience of SMRU working with the population has resulted in a high level of trust in the services. The maternal and child health services are in rural areas, can be provided in Karen and Burmese language, and is without costs [5, 34, 35].

At the time of study protocol submission (August 2017) antiviral treatment of HBV in pregnancy was not provided by the government in Myanmar or in Thailand, although it could be obtained by out of pocket expenditure beyond the means of this marginalized border population.

Study recruitment and enrollment

Pregnant women aged 16-49 years with a singleton viable pregnancy, an HBV infection, and an estimated gestational age of 12 to < 20 weeks determined by ultrasound will be counseled about the disease and invited to participate in the study. Part of this information session will include counseling on HBV in general, the need for adherence to the study drug and benefits and risks of the treatment. If a woman is interested, a blood sample will be collected to determine if she meets the criteria for enrollment i.e. HBeAg positive or HBV

For peer review only - http://bmjopen.bmj.com/site/about/guidelines.xhtml

DNA >1.9 log 10 (>85 IU/mL). Women will be informed of the results at the next visit and be enrolled and started on TDF if all inclusion criteria are met (Table 1).

SMRU has conducted research for over two decades and the trust in antenatal services is high resulting in high follow up rates in ANC as well as in previous studies[36]. If a woman misses an appointment she will be contacted using her phone number if applicable or the healthcare workers will reach out to her community or family to find her.

Follow up

Medical and Obstetric Data

Vital signs (blood pressure, tympanic temperature, heart rate, respiratory rate, fetal heart rate); weight, height, age, symphysis fundal height, fetal gestational age and heartbeat; medical history review (including medications, vitamins and allergies), ethnicity, details of any past pregnancies, complications, place of delivery and outcome will be recorded; and physical examination completed (**Figure 2 and Table 2**).

Laboratory tests

A maternal whole blood sample obtained by venipuncture will be collected at study entry, monthly until delivery, at birth and at two post-partum visits (Figure 2). Maternal HBV DNA, alanine amniotransferase (ALT) and creatinine will be measured at all scheduled visits for the mother. Serum phosphate will be measured at baseline and at birth.

Monthly visits during pregnancy

From the time TDF commences, the pregnant women will return for a monthly study visit. Obstetric follow-up at each monthly visit will include vital signs, weight, symphysis fundal height measurement and fetal heart beat and concomitant medications will be recorded into the CRF. Fetal growth will be measured monthly by ultrasound (head circumference, abdominal circumference and femur length).

Adverse events (AE) will be reviewed, and reported as AE or serious AE (SAE) according to the problem. Compliance will be monitored by pill counting and a questionnaire on adherence.

For peer review only

Delivery

Pregnancy complications and mode of delivery will be recorded in the CRF. Anthropometric measurements of the infant will be done in the first hour of life and will include birth weight, length, head and arm circumference, by trained staff and according to standard operating procedures.

Vaccination: HBIG (if mother is HBeAg positive) and standard HBV vaccine (at birth and 2, 4 and 6 months of age) will be provided to the infants according to the Expanded Program on Immunization (EPI) [37-39].

At birth a 12 ml sample of cord blood, 6 ml into EDTA and 6 ml into a plain tube, will be collected for analysis of HBV DNA and tenofovir level and HBsAg reflecting the HBV status of the infant. Note this cord blood sample might not be collected if the woman is referred for caesarean section into the Public Hospital system.

A maternal serum sample will be used to measure ALT, creatinine, phosphorus, HBsAg (HBeAg only if the woman was HBeAg positive at baseline) and tenofovir level.

Post-partum follow-up

TDF will be continued until one month post-partum to decrease the risk of flares as the maternal immune system returns to baseline. All women will continue the study until the four month post-partum visit. At the 1 and 4 months post-partum visits ALT will be measured. If ALT concentrations are increased, but there is not a true flare, follow up will continue and repeat ALT testing will be done at six months. If the flare worsens or does not resolve, they will be placed on anti-HBV therapy and be reviewed by a physician.

All infants will be followed at 1, 2, 4 and 6 months. The infant will be screened for HBV at two months. At each visit infant anthropometry will be measured (body weight, length, head circumference) and feeding recorded. At six months neurodevelopment will be assessed. At month 2, 4 and 6 the infant will receive follow up HBV vaccinations.

For peer review only - http://bmjopen.bmj.com/site/about/guidelines.xhtml

Laboratory based stopping criteria

ALT: Monthly lab results will be compared to baseline. Grade 3 or higher elevations will be an indication to stop the drug Grade 3 or higher is >5x upper limit of normal (ULN) if baseline levels were below the ULN; or if baseline levels were >ULN and , then grade 3 or higher is >3.5x the baseline value. The upper limit of normal of ALT will be defined as (0-31 U/L) [40].

Creatinine: A creatinine value of more than 1.0 mg/dL will be an indication to stop the drug regardless of the baseline value [40].

Sample size

We calculated the sample size required to estimate the proportion of pregnant women expected to be undetectable at delivery with precision.

$$n = \frac{(1.96)^2 p(1-p)}{d^2}$$

There is no prior knowledge to predict the proportion of pregnant women expected to be undetectable at delivery, so we used the most conservative estimate. When this proportion is 50%, the required sample size is the largest. Based on a 95% confidence interval, a margin of error d = 8% and a conservative estimation of proportion p = 0.5, a sample size of n = 150 is required. We aim for n = 170 to allow for attrition.

Data analyses

HBV Viral kinetics

The Kaplan-Meier method will be used to estimate the median time from initiation of TDF to an HBV DNA <100 IU/ml including all women and then, based on the distribution of HBV DNA, stratified by high vs low HBV DNA groups. We expect those with low HBV DNA at enrolment to have <100 IU/ml at the time of delivery, but it is important to include them in this study to calculate the trajectory of decline and determine how long they need to be treated to reach HBV DNA <100 IU/ml. A Generalized Estimating Equation (GEE) model will be used to assess whether baseline HBV DNA and adherence to TDF are associated with the logarithmically transformed HBV DNA over time. A proportional hazard model will be constructed to assess the hazard ratio of reaching undetectable HBV DNA adjusting for the baseline HBV DNA concentration and adherence to TDF (# pills actually taken/# pills that should have been taken). For missing

For peer review only - http://bmjopen.bmj.com/site/about/guidelines.xhtml

For peer review only

HBV DNA data, depending on the reasons for being missing, imputations using Multiple Imputations by Chained Equations (MICE) will be used. Sensitivity analyses will then be performed comparing analyses using complete and imputed data sets. The proportion of women with undetectable HBV DNA at delivery will be reported with 95% confidence interval in all women and by high vs low HBV DNA groups. Median and interquartile range (IQR) of HBV DNA at baseline and each visit will be reported. Then HBV DNA will be logarithmically transformed and the median HBV DNA at baseline and at delivery will be compared using paired t-tests. Additional analyses may be conducted to further explore the data.

Adherence

The main analysis to determine adherence will be calculating percent adherence using pill counting data (# pills actually taken/# pills that should have been taken). As a confirmation of adherence, we will also measure TDF concentration in all women with detectable HBV DNA at delivery and in a matched subset of women who become undetectable for comparison (n=2x the number of women who do not become undetectable). This is anticipated to include 10-15 women who are HBV DNA detectable at delivery and matched to 20-30 women who are undetectable. This analytic sub-sample will be matched on age and initial DNA concentration or, if a matched pair analysis is not feasible because no "matching partners" exist, we will correct for these factors using multivariate regression methods.

The 'Adherence Starts with Knowledge' questionnaire ASK-12 is a tool to determine adherence and is derived from the ASK-20 questionnaire [41]. The questionnaire consists of 12 items related to drug adherence in three scales "forgetfulness", "treatment beliefs", and "behavior". Although previous studies have demonstrated the usefulness of ASK-12 for patients, this tool has not been validated in this study population [42, 43]. Local pilot testing has resulted in a culturally appropriate version of this questionnaire and personnel will be trained to use it. We will use pill counts (gold standard) as well as TDF blood concentration (confirmatory) to validate this tool. The results will be analysed by first comparing differences among the groups, one-way analysis of variance and Bonferroni's multiple comparison tests.

Hepatic Flare

Although TDF is thought to be safe and acceptable in pregnancy, after discontinuation it might lead to hepatitis flares. There are conflicting data about the proportion and severity of flares after discontinuing antiviral therapy [44-46]. There are no data in pregnant women regarding flares with longer duration of

For peer review only - http://bmjopen.bmj.com/site/about/guidelines.xhtml

treatment and lower HBV DNA at delivery, nor is there information about predisposing characteristics to provide recommendations either before, or even after, flares start. We will determine the proportion of woman who have a moderate (ALT>2.5x upper limit of normal) or severe (ALT>5x upper limit of normal) hepatitis flare after discontinuation of TDF [47]. We will estimate the impact of potential risk factors such as age, number of prior pregnancies, etc. for moderate or severe flares by including treatment duration and HBV DNA concentration at study start and at delivery into multivariable regression models.

Secondary analyses

We will screen for HBV infection by screening for HBsAg in infants at two months of age. If HBsAg is positive, presence of HBV DNA from the same blood sample will be confirmed. If MTCT of HBV exists, the time of TDF initiation, maternal baseline HBV DNA, and TDF adherence will be compared between mothers with MTCT and those without using t-test and Chi-square test/Fisher's exact test. Infant anthropometry will be reported at 1, 2, 4 and 6 months. We will compare the infant anthropometry to the same age infant in general population. Additional analyses such as correlation between TDF use time and infant anthropometry will be conducted based on the data we observe.

ETHICS AND DISSEMINATION

Ethical considerations

SMRU has worked with pregnant women for three decades and provided research results that have had significant local and international impact [30, 48]. Maternal and infant mortality have decreased significantly over the years SMRU has worked with the population. Women come to deliver with SMRU by choice. These marginalized populations and their ability to negotiate the health landscape may be limited by education level but this is recognized by local health staff who are from the same community and form part of the research team [30, 48]. All communication is conducted in the preferred language of the patient and additional efforts are made to assist non-literate women. We ensure that participation is voluntary and that it is clearly understood that participation in the study can be ceased at any time without effect to the care provided. Extra precautions to inform participants about discontinuation while under TDF are also part of the information sheets.

For peer review only - http://bmjopen.bmj.com/site/about/guidelines.xhtml

The Tak Community Advisory Board (T-CAB) reviewed the project for relevance, advice and acceptability in a meeting in relation to this study prior to submission of the protocol [49]. Pertinent responses from the T-CAB in relation to this study were positive but also included a request to include adolescent pregnant women. Several T-CAB members discussed further the issue of consent and assent in relation to SMRU staff, pregnant women, study participants and guardians to further inform the study team on how this issue is best managed with the marginalized, mobile border population.

This study has been approved by the Ethics Committee of the Faculty of Tropical Medicine, Mahidol University (FTM ECF-019-06), Johns Hopkins University (IRB No: 00007432), Chiang Mai University (FAM-2559-04227), Oxford Tropical Research Ethics Committee (OxTREC Reference: 49-16) and by the local Tak Community Advisory Board (TCAB-02/REV/2016).

Informed consent

Trained counsellors will seek the mother's consent after the patient information sheet has been discussed. All queries from the mothers will be answered and those who agree will indicate consent by signing or providing thumbprints. All data collected will be treated confidentially and stored securely.

Patient and Public Involvement statement

The Tak Community Advisory Board (T-CAB) reviewed the project for relevance, advice and acceptability in a meeting in relation to this study prior to submission of the protocol. Pertinent responses from the T-CAB in relation to this study were positive but also included a request to include adolescent pregnant women. Several T-CAB members discussed further the issue of consent and assent in relation to SMRU staff, pregnant women, study participants and guardians to further inform the study team on how this issue is best managed with the marginalized, mobile border population.

For peer review only - http://bmjopen.bmj.com/site/about/guidelines.xhtml

List of abbreviations

AE Adverse event

ANC Antenatal Care

ASK Adherence Starts with Knowledge

DNA deoxyribonucleic acid

HBeAg Hepatitis B e antigen

HBIG Hepatitis B immunoglobulin

HBsAg Hepatitis B surface antigen

HBV Hepatitis B virus

LIC Low-income country

MTCT Mother to child transmission

SMRU Shoklo Malaria Research Unit

TDF Tenofovir Disoproxil Fumarate

Author Statements

KN, SE, CLT and RM designed the protocol; SE, KN and CLT obtained funding; RM, CLT, SE, FT, CA and MB obtained the Ethical approvals; RM and MB set up the clinical work; WW and CLL prepared laboratory testing; NG and YJ supported statistical considerations; PJ, VIC, FT, MvV, MJR and FN supported the design and clinical implementation of the study; all authors read and contributed to the present manuscript. All authors read and approved the final manuscript.

Data statement

After analyses of the data the data will be made available.

Funding

The work is supported by the following awards: Thrasher Research Fund [grant number: TRF13096] to Johns Hopkins University; the Wellcome-Trust Major Overseas Program in Southeast Asia [grant number:

For peer review only - http://bmjopen.bmj.com/site/about/guidelines.xhtml

106698/Z/14/Z] to Mahidol University Oxford Tropical Medicine Research Programme which directly supports PJ, CLL, FN, RM from Shoklo Malaria Research Unit; and Chiang Mai University Thailand supporting MB, FT and CA.

Competing interests

The authors declare that they have no competing interests

Acknowledgements

We would kindly like to acknowledge all contributions of SMRU staff and doctors for their feedback in finalizing the research proposal. Also the data management team for contributing to the Case Report Form development and our quality control team, Moo Kho Paw and Rattanaporn Raksapraidee, and clinical trials support group, for their efforts. Myo Chit Min helped to design the map.

References

1. WHO. Guidelines for the prevention, care and treatment of persons with chronic hepatitis B infection. Geneva: World Health Organization; 2015. Report No.: NLM classification: WC 536.". . Available at: https://www.who.int/hepatitis/publications/hepatitis-b-guidelines/en/. Accessed 19 December 2019.

2. Thomas DL. Global Elimination of Chronic Hepatitis. N Engl J Med **2019**; 380(21): 2041-50.

3. Bloom DE, Khoury A, Subbaraman R. The promise and peril of universal health care. Science (New York, NY) **2018**; 361(6404).

4. Banks T, Kang J, Watts I, et al. High hepatitis B seroprevalence and risk factors for infection in pregnant women on the Thailand-Myanmar Border. J Infect Dev Ctries **2016**; 10(4): 384-8.

5. Bierhoff M, Angkurawaranon C, Myat Min A, et al. Maternal Hepatitis B Infection Burden, Comorbidity and Pregnancy Outcome in a Low-Income Population on the Myanmar-Thailand Border: A Retrospective Cohort Study. J Pregnancy **2019**; 2019: 8435019.

6. Pan CQ, Duan Z, Dai E, et al. Tenofovir to Prevent Hepatitis B Transmission in Mothers with High Viral Load. N Engl J Med **2016**; 374(24): 2324-34.

For peer review only - http://bmjopen.bmj.com/site/about/guidelines.xhtml

7. Thio CL, Guo N, Xie C, Nelson KE, Ehrhardt S. Global elimination of mother-to-child transmission of hepatitis B: revisiting the current strategy. Lancet Infect Dis **2015**; 15(8): 981-5.

8. Lattof SR, Tuncalp O, Moran AC, et al. Developing measures for WHO recommendations on antenatal care for a positive pregnancy experience: a conceptual framework and scoping review. BMJ open **2019**; 9(4): e024130.

9. Li XM, Yang YB, Hou HY, et al. Interruption of HBV intrauterine transmission: a clinical study. World J Gastroenterol **2003**; 9(7): 1501-3.

10. Beigi R, Noguchi L, Parsons T, et al. Pharmacokinetics and placental transfer of single-dose tenofovir 1% vaginal gel in term pregnancy. J Infect Dis **2011**; 204(10): 1527-31.

11. Thilakanathan C, Wark G, Maley M, et al. Mother-to-child transmission of hepatitis B: Examining viral cut-offs, maternal HBsAg serology and infant testing. Liver international : official journal of the International Association for the Study of the Liver **2018**; 38(7): 1212-9.

12. Wen WH, Chang MH, Zhao LL, et al. Mother-to-infant transmission of hepatitis B virus infection: significance of maternal viral load and strategies for intervention. J Hepatol **2013**; 59(1): 24-30.

13. Lu Y, Zhu FC, Liu JX, et al. The maternal viral threshold for antiviral prophylaxis of perinatal hepatitis B virus transmission in settings with limited resources: A large prospective cohort study in China. Vaccine **2017**; 35(48 Pt B): 6627-33.

14. Jourdain G, Ngo-Giang-Huong N, Harrison L, et al. Tenofovir versus Placebo to Prevent Perinatal Transmission of Hepatitis B. New England Journal of Medicine **2018**; 378(10): 911-23.

15. Lee YS, Lee HS, Kim JH, et al. Role of tenofovir disoproxil fumarate in prevention of perinatal transmission of hepatitis B virus from mother to child: a systematic review and meta-analysis. The Korean journal of internal medicine **2019**.

16. Gao F, Zhang WT, Lin YY, Wang WM, Xu N, Bai GQ. Early Start Of Tenofovir Treatment Achieves Better Viral Suppression In Pregnant Women With A High HBV Viral Load: A Real-World Prospective Study. Infection and drug resistance **2019**; 12: 3475-84.

17. Thio CL, Guo N, Xie C, Nelson KE, Ehrhardt S. Global elimination of mother-to-child transmission of hepatitis B: revisiting the current strategy. The Lancet Infectious Diseases **2015**; 15(8): 981-5.

For peer review only

For peer review only - http://bmjopen.bmj.com/site/about/guidelines.xhtml

18. Colbers AP, Hawkins DA, Gingelmaier A, et al. The pharmacokinetics, safety and efficacy of tenofovir and emtricitabine in HIV-1-infected pregnant women. AIDS (London, England) **2013**; 27(5): 739-48.

19. Pyra M, Anderson PL, Hendrix CW, et al. Tenofovir and tenofovir-diphosphate concentrations during pregnancy among HIV-uninfected women using oral preexposure prophylaxis. AIDS (London, England) **2018**; 32(13): 1891-8.

20. Bierhoff M, Pinyopornpanish K, Pinyopornpanish K, et al. Retrospective Review of Documentation Practices of Hepatitis B Immunoglobulin, Birth Dose, and Vaccination at the Hospital of Birth, in Thai Nationals and Migrants in Northern Thailand. Open forum infectious diseases **2019**; 6(12): ofz518.

21. Best BM, Burchett S, Li H, et al. Pharmacokinetics of tenofovir during pregnancy and postpartum. HIV Med **2015**; 16(8): 502-11.

22. Benaboud S, Hirt D, Launay O, et al. Pregnancy-related effects on tenofovir pharmacokinetics: a population study with 186 women. Antimicrob Agents Chemother **2012**; 56(2): 857-62.

23. Bierhoff M, Smolders EJ, Tarning J, et al. Pharmacokinetics of oral tenofovir disoproxil fumarate in pregnancy and lactation: a systematic review. Antiviral therapy **2019**; 24(7): 529-40.

24. Cressey TR, Avihingsanon A, Halue G, et al. Plasma and Intracellular Pharmacokinetics of Tenofovir Disoproxil Fumarate 300 mg Every 48 Hours vs 150 mg Once Daily in HIV-Infected Adults With Moderate Renal Function Impairment. Clin Infect Dis **2015**; 61(4): 633-9.

25. Siberry GK, Jacobson DL, Kalkwarf HJ, et al. Lower Newborn Bone Mineral Content Associated With Maternal Use of Tenofovir Disoproxil Fumarate During Pregnancy. Clin Infect Dis **2015**; 61(6): 996-1003.

26. Van Dyke RB, Chadwick EG, Hazra R, Williams PL, Seage GR, 3rd. The PHACS SMARTT Study: Assessment of the Safety of In Utero Exposure to Antiretroviral Drugs. Front Immunol **2016**; 7: 199.

27. Yankam BM, Anye CS, Nkfusai NC, Shirinde J, Cumber SN. Knowledge and practice of pregnant women and health care workers on hepatitis B prevention in the Limbe and Muyuka health districts of the south west region of Cameroon. The Pan African medical journal **2019**; 33: 310.

28. Durante-Mangoni E, Vitrone M, Parrella A, et al. Efficacy and safety of tenofovir, entecavir, and telbivudine for chronic hepatitis B in heart transplant recipients. Transpl Infect Dis **2016**; 18(3): 319-25.

For peer review only

29. Suk-Fong Lok A. Hepatitis B Treatment: What We Know Now and What Remains to Be Researched. Hepatology communications **2019**; 3(1): 8-19.

30. Gilder ME, Moo P, Hashmi A, et al. "I can't read and don't understand": Health literacy and health messaging about folic acid for neural tube defect prevention in a migrant population on the Myanmar-Thailand border. PLoS One **2019**; 14(6): e0218138.

31. Manoyos V TA, Srithanaviboonchai K, Yangyuenkul S, Grimes RM. Sexual risk-behaviors for HIV infections among young cross-border migrant workers living in urban Chiang Mai, Thailand. Journal of Health Research **2016**; 30(5): 347-53.

32. International, Organization, for, Imigration. IOM Thailand National Strategy 2017-2020. Available at: https://thailand.iom.int/iom-thailand-national-strategy-2017-2020. Accessed June 2019.

33. Meyer SR, Decker MR, Tol WA, Abshir N, Mar AA, Robinson WC. Workplace and security stressors and mental health among migrant workers on the Thailand-Myanmar border. Social psychiatry and psychiatric epidemiology **2016**; 51(5): 713-23.

34. Culhane-Pera KA, Sriphetcharawut S, Thawsirichuchai R, Yangyuenkun W, Kunstadter P. Afraid of Delivering at the Hospital or Afraid of Delivering at Home: A Qualitative Study of Thai Hmong Families' Decision-Making About Maternity Services. Matern Child Health J **2015**; 19(11): 2384-92.

35. Suphanchaimat R, Putthasri W, Prakongsai P, Tangcharoensathien V. Evolution and complexity of government policies to protect the health of undocumented/illegal migrants in Thailand - the unsolved challenges. Risk Manag Healthc Policy **2017**; 10: 49-62.

36. Fellmeth G, Plugge EH, Carrara V, et al. Migrant perinatal depression study: a prospective cohort study of perinatal depression on the Thai-Myanmar border. BMJ open **2018**; 8(1): e017129.

37. WHO. Expanded Program on Immunization Factsheet Myanmar. Available at: http://www.searo.who.int/immunization/data/myanmar.pdf. Accessed December 2018.

38. Cutts FT, Claquin P, Danovaro-Holliday MC, Rhoda DA. Monitoring vaccination coverage: Defining the role of surveys. Vaccine **2016**; 34(35): 4103-9.

39. Schalm SW, Mazel JA, de Gast GC, et al. Prevention of hepatitis B infection in newborns through mass screening and delayed vaccination of all infants of mothers with hepatitis B surface antigen. Pediatrics **1989**; 83(6): 1041-8.

For peer review only - http://bmjopen.bmj.com/site/about/guidelines.xhtml

40. Abbassi-Ghanavati M, Greer LG, Cunningham FG. Pregnancy and laboratory studies: a reference table for clinicians. Obstet Gynecol **2009**; 114(6): 1326-31.

41. Matza LS, Park J, Coyne KS, Skinner EP, Malley KG, Wolever RQ. Derivation and validation of the ASK-12 adherence barrier survey. The Annals of pharmacotherapy **2009**; 43(10): 1621-30.

42. Sasaki J, Kawayama T, Yoshida M, et al. Adherence barriers to inhaled medicines in Japanese older patients with asthma evaluated using the "Adherence Starts with Knowledge 20" (ASK-20) questionnaire. The Journal of asthma : official journal of the Association for the Care of Asthma **2019**; 56(6): 632-41.

43. Kimura Y, Koya T, Hasegawa T, et al. Characterization of low adherence population in asthma patients from Japan using Adherence Starts with Knowledge-12. Allergology international : official journal of the Japanese Society of Allergology **2020**; 69(1): 61-5.

44. Nguyen V, Tan PK, Greenup AJ, et al. Anti-viral therapy for prevention of perinatal HBV transmission: extending therapy beyond birth does not protect against post-partum flare. Aliment Pharmacol Ther **2014**; 39(10): 1225-34.

45. Xu WM, Cui YT, Wang L, et al. Lamivudine in late pregnancy to prevent perinatal transmission of hepatitis B virus infection: a multicentre, randomized, double-blind, placebo-controlled study. Journal of viral hepatitis **2009**; 16(2): 94-103.

46. Liem KS, Gehring AJ, Feld JJ, Janssen HLA. Challenges with stopping long-term nucleos(t)ide analogue therapy in patients with chronic hepatitis B. Gastroenterology **2019**.

47. Wang L, Kourtis AP, Ellington S, Legardy-Williams J, Bulterys M. Safety of tenofovir during pregnancy for the mother and fetus: a systematic review. Clin Infect Dis **2013**; 57(12): 1773-81.

48. Parker DM, Carrara VI, Pukrittayakamee S, McGready R, Nosten FH. Malaria ecology along the Thailand-Myanmar border. Malar J **2015**; 14: 388.

49. Pratt B, Lwin KM, Zion D, Nosten F, Loff B, Cheah PY. Exploitation and community engagement: can community advisory boards successfully assume a role minimising exploitation in international research? Dev World Bioeth **2015**; 15(1): 18-26.

For peer review only - http://bmjopen.bmj.com/site/about/guidelines.xhtml

For peer review only

For peer review only - http://bmjopen.bmj.com/site/about/guidelines.xhtml

Figure 1. Map of the central office of SMRU in Mae Sot, and the study sites Wang Pha (WPA), Mawker Thai (MKT) and Maela (MLA) on the Myanmar-Thailand border where the study will be conducted.

For peer review only

For peer review only - http://bmjopen.bmj.com/site/about/guidelines.xhtml

Figure 2. Schematic reproduction of the study design

ALT, Alanine Aminotransferase; Cr, Creatinine; DNA, deoxyribonucleic acid; EGA, estimated gestational age; HBeAg, Hepatitis B envelope antigen; HBIG, Hepatitis B immunoglobulins; HBsAg, Hepatitis B surface antigen; HBV, Hepatitis B virus; TDF, Tenofovir Disoproxil Fumarate

For peer review only

For peer review only - http://bmjopen.bmj.com/site/about/guidelines.xhtml

Table 1. Inclusion and exclusion criteria

Inclusion criteria	Exclusion criteria
Willing and able to give informed consent for participation in the study	Negative qualitative HBV DNA if HBeAg negative
Hepatitis B infected (HBsAg confirmed positive and qualitative HBV DNA positive in HBeAg negative mothers)	HIV positive or on immunosuppressive therapy for other illnesses
Female, 16-49 years	Elevated creatinine (>1 mg/dL)
EGA[1] 12-20 weeks at start TDF[2]	Abnormal serum phosphate (<2.4 and >4.5 mg/dL)
Willing to take TDF[2] daily during pregnancy	History of kidney disease
Plans to deliver at SMRU[3]	History of pregnancy complications

[1]Estimated Gestational Age, [2]Tenofovir Disoproxil Fumarate, [3]Shoklo Malaria Research Unit

Table 2. Overview of measurements and interventions per visit

Procedures	Visits timing						
	Start	Monthly visits	Delivery	1 month PP	2 months PP	4 months PP	6 months PP
		On TDF	On TDF	Stop TDF		End mother	End infant
Eligibility checklist	X						
Informed consent	X						
Ultrasound	X	X	X				
Demographics	X						
Medical history	X						
Physical examination	X	X	X	X		X	
**Laboratory tests**							
HBsAg/HBeAg	X						
HBV DNA	X	X	X	X			
Creatinine	X	X	X	X			
Serum Phosphate	X		X				
ALT	X	X	X	X		X[1]	
TDF Concentration			X[2]				
Cord HBsAg/HBV DNA			X[2]				

For peer review only - http://bmjopen.bmj.com/site/about/guidelines.xhtml

Infant HBsAg					X[4]		
Pill count		X	X	X			
Adverse event assessments		X	X	X			
Infant anthropometry			X	X	X	X	X
Infant neurodevelopment							X
Adherence survey		X	X	X			
Infant HB vaccination			X[3]		X	X	X

[1] Follow-up will continue if suspected or confirmed flare, [2] Cord blood sample, [3] If mother HBeAg positive also HBIG , [4] Venepuncture sample blood and only for HBsAg

For peer review only

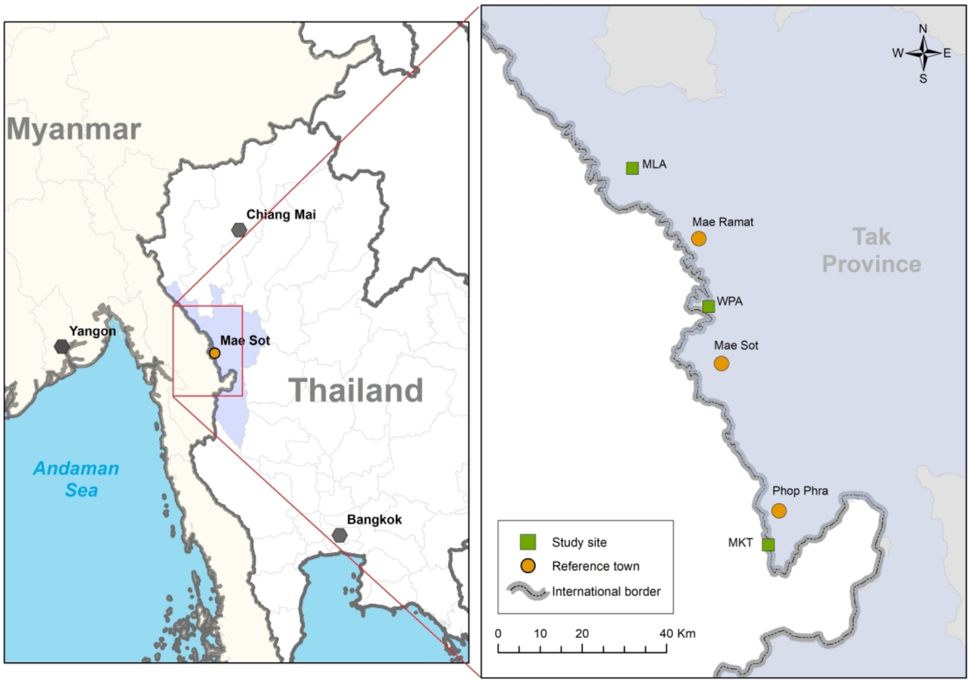

Figure 1. Map of the central office of SMRU in Mae Sot, and the study sites Wang Pha (WPA), Mawker Thai (MKT) and Maela (MLA) on the Myanmar-Thailand border where the study will be conducted.

296x210mm (400 x 400 DPI)

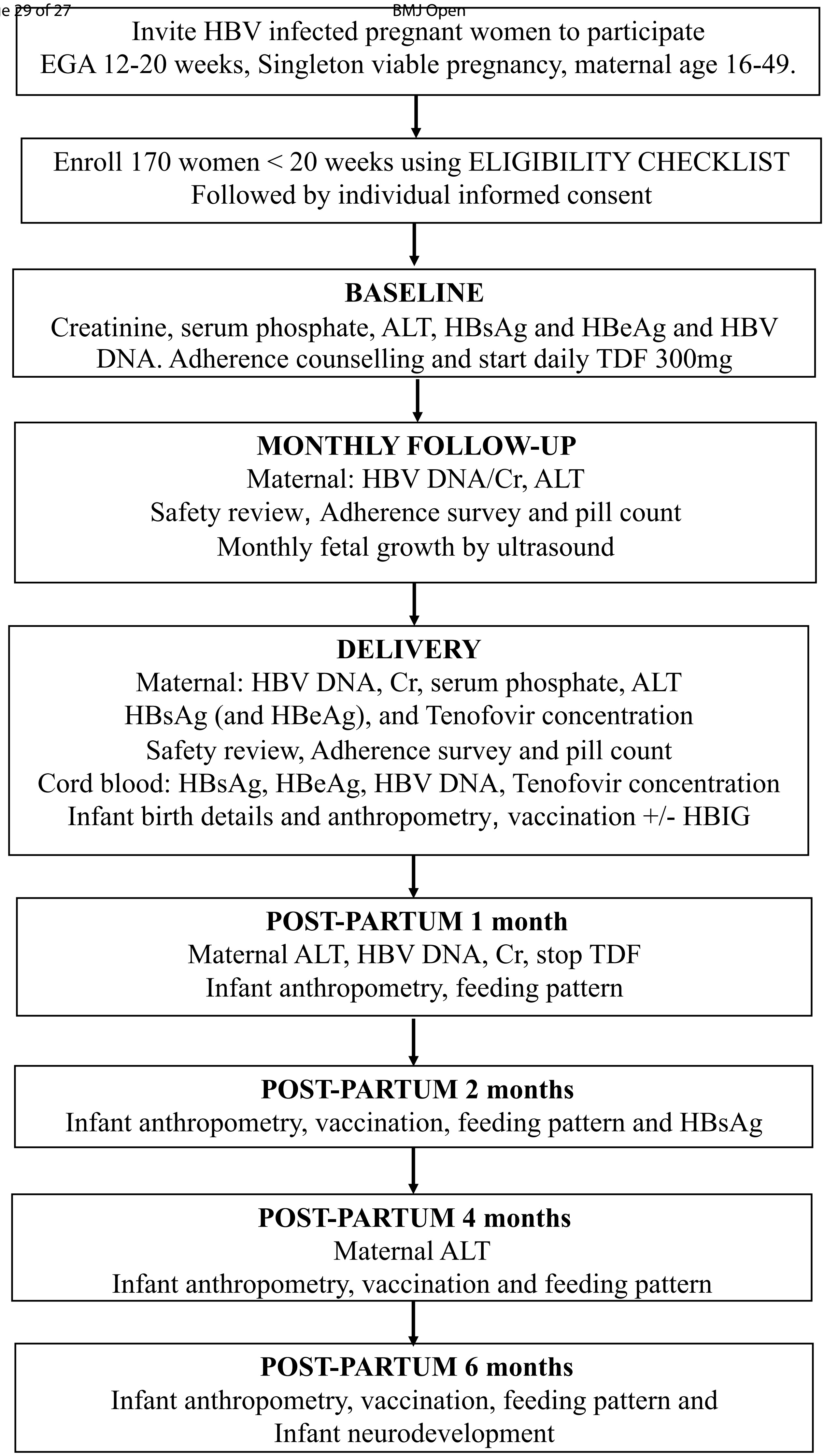
Invite HBV infected pregnant women to participate
EGA 12-20 weeks, Singleton viable pregnancy, maternal age 16-49.
Enroll 170 women < 20 weeks using ELIGIBILITY CHECKLIST
Followed by individual informed consent
BASELINE
Creatinine, serum phosphate, ALT, HBsAg and HBeAg and HBV DNA. Adherence counselling and start daily TDF 300mg
MONTHLY FOLLOW-UP
Maternal: HBV DNA/Cr, ALT
Safety review, Adherence survey and pill count
Monthly fetal growth by ultrasound
DELIVERY
Maternal: HBV DNA, Cr, serum phosphate, ALT
HBsAg (and HBeAg), and Tenofovir concentration
Safety review, Adherence survey and pill count
Cord blood: HBsAg, HBeAg, HBV DNA, Tenofovir concentration
Infant birth details and anthropometry, vaccination +/- HBIG
POST-PARTUM 1 month
Maternal ALT, HBV DNA, Cr, stop TDF
Infant anthropometry, feeding pattern
POST-PARTUM 2 months
Infant anthropometry, vaccination, feeding pattern and HBsAg
POST-PARTUM 4 months
Maternal ALT
Infant anthropometry, vaccination and feeding pattern
POST-PARTUM 6 months
Infant anthropometry, vaccination, feeding pattern and
Infant neurodevelopment
